# The Impact of a Nickel-Copper Smelter on Concentrations of Toxic Elements in Local Wild Food from the Norwegian, Finnish, and Russian Border Regions

**DOI:** 10.3390/ijerph14070694

**Published:** 2017-06-28

**Authors:** Martine D. Hansen, Therese H. Nøst, Eldbjørg S. Heimstad, Anita Evenset, Alexey A. Dudarev, Arja Rautio, Päivi Myllynen, Eugenia V. Dushkina, Marta Jagodic, Guttorm N. Christensen, Erik E. Anda, Magritt Brustad, Torkjel M. Sandanger

**Affiliations:** 1 Department of Community Medicine, Faculty of Health Sciences, UiT—The Arctic University of Norway, NO-9037 Tromsø, Norway; therese.h.nost@uit.no (T.H.N.); erik.anda@uit.no (E.E.A.); magritt.brustad@uit.no (M.B.); torkjel.sandanger@uit.no (T.M.S.); 2 NILU—Norwegian Institute for Air Research, The Fram Centre, NO-9296 Tromsø, Norway; esh@nilu.no; 3 Akvaplan-niva, The Fram Centre, NO-9296 Tromsø, Norway; Anita.Evenset@akvaplan.niva.no (A.E.); guttorm.christensen@akvaplan.niva.no (G.N.C.); 4 Faculty of Biosciences, Fisheries and Economics, UiT—The Arctic University of Norway, NO-9037 Tromsø, Norway; 5 Hygiene Department, Northwest Public Health Research Centre (NWPHRC), St. Petersburg 191036, Russia; alexey.d@inbox.ru (A.A.D.); dushka9005@mail.ru (E.V.D.); 6 Arctic Health, Faculty of Medicine and Thule Institute, University of Oulu, FI-90014 Oulu, Finland; arja.rautio@oulu.fi; 7 Northern Laboratory Centre NordLab, FI-90220 Oulu, Finland; paivi.myllynen@nordlab.fi; 8 Department of Environmental Sciences, Jožef Stefan Institute, 1000 Ljubljana, Slovenia; marta.jagodic@ijs.si

**Keywords:** toxic elements, nickel smelter, metallurgic industry, local food, berries, mushrooms, environmental pollution, Kola Peninsula, Arctic

## Abstract

Toxic elements emitted from the Pechenganickel complex on the Kola Peninsula have caused concern about potential effects on local wild food in the border regions between Norway, Finland and Russia. The aim of this study was to assess Ni, Cu, Co, As, Pb, Cd, and Hg concentrations in local wild foods from these border regions. During 2013–2014, we collected samples of different berry, mushroom, fish, and game species from sites at varying distances from the Ni-Cu smelter in all three border regions. Our results indicate that the Ni-Cu smelter is the main source of Ni, Co, and As in local wild foods, whereas the sources of Pb and Cd are more complex. We observed no consistent trends for Cu, one of the main toxic elements emitted by the Ni-Cu smelter; nor did we find any trend for Hg in wild food. Concentrations of all investigated toxic elements were highest in mushrooms, except for Hg, which was highest in fish. EU maximum levels of Pb, Cd, and Hg were exceeded in some samples, but most had levels considered safe for human consumption. No international thresholds exist for the other elements under study.

## 1. Introduction

Point-source pollution due to toxic elements remains a challenge to the environment and to humans in some areas of the world. Although most point-source pollution is situated at lower latitudes, there is some point-source pollution in the Arctic and sub-Arctic regions. One example is the Pechenganickel complex on the Russian Kola Peninsula, which is located close to the border between Norway, Finland, and Russia. The Pechenganickel complex, located in the Russian city of Nikel, close to the Norwegian border, along with the briquetting facility in the city of Zapolyarny, located approximately 25 km from the smelter, comprises one of the largest nickel (Ni)–copper (Cu) smelters in the world [1,2,3,4]. The complex as a whole has been emitting large amounts of Ni, Cu, cobalt (Co), sulphur dioxide (SO_2_), and dust particles into the atmosphere and local waters since Ni-Cu smelting began in the 1930s [1,3,4,5,6,7,8,9,10,11]. Although trace amounts of Cu are essential to humans, larger amounts can be hazardous [12]. Other toxic elements, such as arsenic (As), lead (Pb), cadmium (Cd), and mercury (Hg) are also emitted by the complex, but studies disagree on the magnitude of these emissions [1,3,4,5,6,7,10,13,14]. It is also likely that As, Pb, Cd, and Hg emissions are camouflaged by deposition from long-range transport and other local anthropogenic or geogenic sources [3,4,5]. According to the latest estimates, the Pechenganickel complex emitted 330 tons of Ni and 158 tons of Cu into the atmosphere in 2009, and these numbers have remained unchanged since the 1990s [1]. A modernization of the briquette facility in the city of Zapolyarny was completed in 2015, which may reduce emissions from this part of the complex [1]. 

There are seasonal differences in wind patterns in the border regions between Norway, Finland, and Russia. The prevailing winds are primarily from the south and south-east during the winter months and from all directions during the summer months [1,15]. Based on this, it is believed that airborne pollution is mainly transported to the border regions between Norway and Russia, north of the smelter. Although not in the direction of the prevailing winds, a previous study has suggested that Ni and Cu emissions from the Pechenganickel complex may even travel as far as Greenland [16]. In addition to airborne pollution, wastewater discharge from the Ni-Cu smelter, as well as from the city of Nikel, conveys toxic elements into Kuetsjarvi Lake, located in the Pasvik watercourse (forming the border between Norway and Russia) close to the smelter [13,17,18,19].

The border regions between Norway, Finland, and Russia are fairly densely populated. The highest population density is on the Russian side of the Norwegian-Russian border, with 4.5 inhabitants per km^2^. In 2015, approximately 39,000 people lived in the Pechenga district in Murmansk Oblast, Russia, and 3/4 of these lived in the cities of Nikel and Zapolyarny. In the Norwegian municipality of Sør-Varanger, which borders Russia and Finland, the population density is 2.5 inhabitants per km^2^ and approximately 10,000 people resided there in 2015 [20]. The least densely populated area is the municipality of Inari on the Finnish side of the border, with a population of approximately 7000. The population density in this municipality is 0.41 inhabitants per km^2^ [21].

Elevated environmental concentrations of toxic elements such as Ni, Cu, and several others in these border regions, as well as several air pollution events [1,2,3,4,5,6,9,10,13,14,18,22,23,24,25,26,27], have given rise to concern among these residents [27], some of whom frequently use foods that grow wild in these regions. Therefore, knowledge of the concentrations of toxic elements in local wild foods is important, both to protect nature and to protect humans that consume local wild food. 

As a part of the trilateral KolArctic project “Food and Health Security in the Norwegian, Russian and Finnish border regions: linking local industries, communities and socio-economic impacts”, the overall aim of this study was to assess the impact of emissions from the Ni-Cu smelter in the Pechenganickel complex on Ni, Cu, Co, As, Pb, Cd, and Hg concentrations in commonly used local wild foods. 

## 2. Materials and Methods

### 2.1. Sampling Sites and Sample Collection

**Figure 1:**
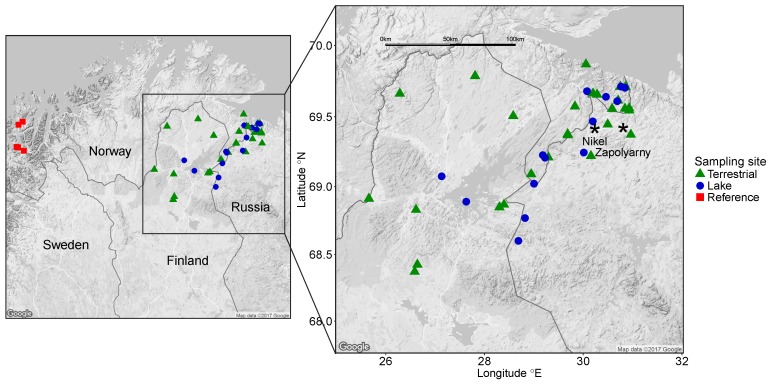
Map of the sampling areas. Terrestrial sites (green triangles and red squares), lakes (blue dots), reference sites not affected by anthropogenic or geogenic pollution (red squares), and the cities of Nikel and Zapolyarny (black stars).

We chose sampling sites that are used frequently for gathering wild berries and mushrooms, fishing, and hunting. Moreover, sampling sites located northeast of the Ni-Cu smelter were prioritized since they are in the path of the prevailing winds. Thus, we included a total of 41 sampling sites in the border regions between Norway, Finland, and Russia (Figure 1). Twenty-one were located in Norway (14 terrestrial sites and seven lakes) 19–57 km north, west, or northeast of the Ni-Cu smelter, 11 in Finland (nine terrestrial sites and two lakes) 91–189 km south, west, and southwest of the Ni-Cu smelter, and nine in Russia (four terrestrial sites and five lakes) 7–110 km north, east, south, and southwest of the Ni-Cu smelter (Table 1 and Table S1). We also collected berries and mushrooms at five sampling sites in Troms County, Norway, 430–445 km west of the Ni-Cu smelter. These sites are not affected by anthropogenic or geogenic pollution and served as reference sites in this study (Figure 1 and Table S1). 

Standardized protocols for sample collection were developed and distributed to the field personnel in all three countries. Most samples were pooled, but some fish and game samples consisted of only one specimen. All samples, pooled or single, weighed between 146 and 770 g. The authors performed most of the sample collection, but in Norway and Finland some samples were obtained from local people who had collected relevant species for their own consumption. 

Berries and mushrooms were handpicked as per local custom. Fish were caught using gillnets. Their length and weight were measured and a sample of muscle tissue was kept for analysis, since this is the tissue most frequently used for human consumption. Fish samples were generally pooled, with 4–10 fish specimens in each sample, but some samples came from individual specimens (Table S1). Reindeer samples from Norway and Finland were obtained from slaughterhouses. All of the reindeer and moose samples were individual samples of neck muscle tissue. We had three individual samples of muscle tissue from Norwegian moose, which were obtained from local hunters. Finally, we had three pooled samples of ptarmigans trapped in Finland that contained breast meat from two, three and four birds, respectively.

Most samples were collected during 2013–2014, save the individual moose samples from Norway, which were collected in 2009, 2010, and 2011. All samples were stored in plastic bags or containers and kept at −20 °C until analysis. Weight, age, and sex were recorded for most of the sampled animals. 

### 2.2. Chemical Analyses 

Berries and mushrooms were not rinsed prior to chemical analysis since the local people normally do not rinse them before consumption. In this way, we aimed to better estimate the true concentrations the inhabitants are exposed to through ingestion. All samples were analyzed on wet weight (w.w.) basis. 

Norwegian samples were analyzed at the laboratory of the Norwegian Institute for Air Research (NILU) in Kjeller, Norway. All samples were prepared in a similar manner: they were digested by microwave-assisted mineralization using an UltraClave (Milestone, Sorisole, Italy). About 0.5–0.75 g of the sample was placed in a TFM tube with 5 mL of diluted supra pure HNO_3_. After digestion, the samples were split into two aliquots. Concentrated supra pure HCl was added to the aliquot to assess Hg concentration. The toxic elements were analyzed by inductively coupled plasma mass spectrometry (ICP-MS) on an Agilent 7700× device (Agilent, Santa Clara, CA, USA). All chemical analyses followed international requirements for quality assurance/control, as recommended by AMAP: the Arctic Monitoring and Assessment Programme, and the requirements in the European quality norm EN 17049. For quality assurance/control purposes, certified reference material was used: SRM-1566b (Oyster tissue, National Institute of Standards and Technology (NIST), Gaithersburg, MD, USA). 

Finnish samples were analyzed in two separate laboratories. Ni concentration was measured at the NILU laboratory, Kjeller, Norway, following the same procedure as for Norwegian samples. Assessment of all other elements in Finnish samples was performed at the Jozef Stefan Institute in Ljubljana, Slovenia. For analysis of Cu, As, Pb, and Cd concentrations, 1 g of homogenized sample was placed in a quartz tube. Three milliliters of 65% supra pure HNO_3_ and 1 mL 30% supra pure H_2_O_2_ were added, and samples were subjected to closed vessel microwave digestion at maximum power of 1500 W, ramped to 130 °C for 10 min, ramped to 200 °C for 10 min, held for 20 min, and then cooled for 20 min. Digested samples were diluted to 20 mL. Measurements were made by an Octapole Reaction System ICP-MS (7500ce, Agilent, Tokyo, Japan). Hg concentration was determined using cold vapor atomic fluorescence spectrometry after wet decomposition of the sample. One gram of homogenized sample was placed into a 100 mL volumetric flask, 2 mL of a mixture of 65% HNO_3_-HClO_4_ (1:1, *v*/*v*), and 5 mL of 96% H_2_SO_4_ were added. The flask was heated at 220 °C on a hotplate for 20 min. Digested samples were diluted to 100 mL and analyzed using the Tekran 2600 Instrument (Tekran, Toronto, ON, Canada) (EPA method 1630). For quality assurance/control purposes, certified reference materials were used: SRM-1570a (Trace elements in Spinach Leaves, NIST), SRM-1573a (Tomato leaves, NIST), SRM-2976 (Muscle Tissue, NIST) and DORM-4 (Fish protein, National Research Council Canada, Measurement Science and Standards, Ottawa, ON, Canada).

Russian samples were analyzed at Typhoon Laboratory (St. Petersburg, Russia). Before analysis, samples were thawed, homogenized, and mineralized. An aliquot of homogenized sample of approximately 1.5 g was weighed. Mineralization was carried out by supra pure HNO_3_, pre-distilled in a DistillAcid apparatus (Berghof, Eningen, Germany) in Teflon tubes in a MARS-5 microwave oven (CEM corporation, Matthews, NC, USA). Samples were exposed at 80 °C for 20 min then heated to 120 °C for 75 min with pressure control. The mineralized sample was diluted with high purity water to a volume of 25 mL. Analysis of mineralized food samples for toxic elements was carried out by ICP-MS on a NexION-300D device (PerkinElmer, Chicago, IL, USA). Instrument calibration was performed on multicomponent standard solutions ICP-MESS-4 and ICP-MESS-16 (Certipur, Merck Chemicals GmbH, Darmstadt, Germany). An additive of yttrium (^89^Y), obtained by dissolving the metal oxide in HNO_3_ of high purity, was used as the internal standard. The analysis of the toxic elements was carried out in the kinetic energy discrimination, Dynamic Reaction Cell™, and normal modes. For quality assurance/control purposes, samples for international intercalibrations under the Northern Contaminants Program quality assurance/control were used. For the certified reference materials, all values were within ±20% of the reference values.

### 2.3. Data Treatment and Statistical Analyses

Spatial trend analyses were performed for individual species and toxic elements only when the number of data points was sufficient to establish a spatial trend or to compare locations. We assessed differences between foods by grouping the individual species into four food groups, i.e., berries, mushrooms, fish, and game. We substituted laboratory values below the limit of detection (LOD) with LOD/$\sqrt{2}$ [28] and this value was used in evaluation of trends and food group differences. Statistical analyses were only performed if more than 80% of the samples in each food group had concentrations above LOD. No further quality assurance/control was used in the statistical analyses.

When more than one sample was collected from the same sampling site, the mean concentration was used in the analyses. STATA (version 14.0, STATA Corp., College Station, TX, USA) and R (version 3.2.1, R Core Development Team, Vienna, Austria) were used for statistical analyses. The Kruskal-Wallis test and Dunn’s test were used to assess differences in concentrations between food groups. Maps were created with the R package ggmaps for visualization of the spatial distribution of concentrations [29]. Principal component analyses were performed to further explore food-specific differences and spatial distributions. The level of significance was originally set at 0.05, but after correction for multiple comparisons by the Simes method [30], it was reduced to 0.03. 

## 3. Results

The total dataset consisted of 174 data points (including reference samples). Few samples were below LOD, except in Hg analyses, in which more than 80% of berry samples were below LOD (see Table S1, where summaries of concentrations are also presented). 

### 3.1. Spatial Trends in Local Wild Foods

#### 3.1.1. Spatial Trends in Berries and Mushrooms

**Figure 2:**
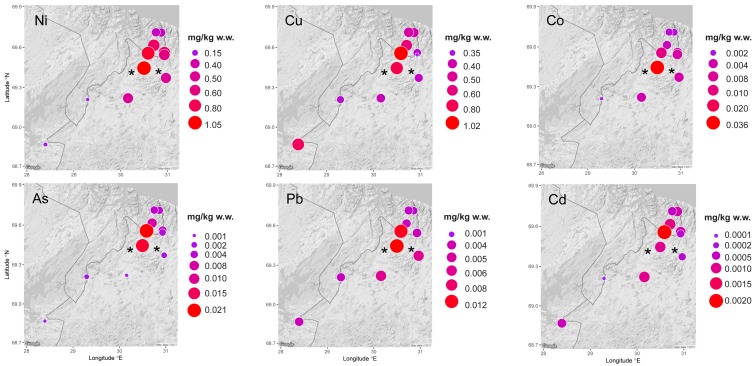
Mean Ni, Cu, Co, As, Pb, and Cd concentrations (mg/kg w.w.) in bilberries in the border regions between Norway, Finland, and Russia. The cities of Nikel and Zapolyarny are indicated by black stars. Note that concentration scales are different for the different toxic elements. w.w.: wet weight.

**Figure 3:**
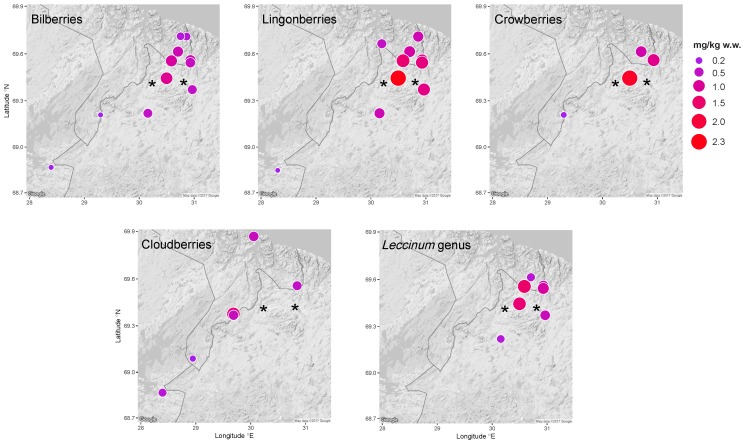
Mean Ni concentrations (mg/kg w.w.) in bilberries, lingonberries, crowberries, cloudberries, and mushrooms (*Leccinum* genus) in the border regions between Norway, Finland, and Russia. The cities of Nikel and Zapolyarny are indicated by black stars.

Ni, Cu, Co, As, Pb, and Cd concentrations were highest in bilberries, lingonberries, and crowberries from sites closest to the Ni-Cu smelter (Figure 2, Figures S1 and S2). The same was true for Ni, Cu, Co, and As concentrations in mushrooms (*Leccinum* genus) (Figure S3). However, Pb and Cd concentrations in lingonberries were equally high in samples collected further from the Ni-Cu smelter. Similar, conflicting results were observed for Cu in mushrooms: the sample from the second-closest site to the Ni-Cu smelter had the highest concentration, but the one from the closest site had the lowest. Elevated Ni concentrations were observed in bilberries, lingonberries, crowberries and mushrooms (*Leccinum* genus) close to the Ni-Cu smelter (Figure 3). 

No clear spatial trend could be established for Ni (Figure 3) or any other element in cloudberries due to varying concentrations as well as scattered samples in relation to the Ni-Cu smelter. 

**Figure 4:**
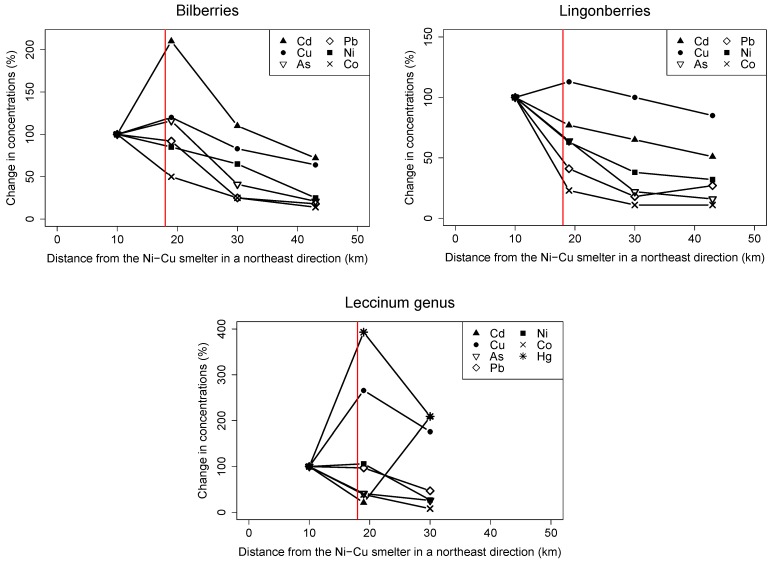
Percent change in Ni, Cu, Co, As, Pb, and Cd concentrations for bilberries and lingonberries, and Ni, Cu, Co, As, Pb, Cd, and Hg concentrations in mushrooms (*Leccinum* genus) as a function of distance from the Ni-Cu smelter for sampling sites located in a northeast direction relative to the site closest to the Ni-Cu smelter (i.e., the Nikel site, 10 km east of the smelter). Vertical line indicates the Norwegian-Russian border. Except for the site closest to the Ni-Cu smelter, all sampling sites were located in Norway. Reference sites are not included in this figure.

The distribution and the higher number of sampling sites northeast of the Ni-Cu smelter allowed us to evaluate changes in concentrations with increasing distance. Generally, concentrations were lower at the site furthest from the smelter, excluding reference sites (43 km for berries and 30 km for mushrooms) than at the site closest to the smelter (Figure 4). This was most pronounced for Ni, Co, As, and Pb in bilberries, lingonberries, and mushrooms (*Leccinum* genus), for which concentrations at the furthest sites were 8–47% of those at the closest site. The difference was less pronounced for Cu and Cd in bilberries and lingonberries, in which concentrations at the furthest sites were 51–85% of those at the closest site. However, Cu, As, and Cd concentrations in bilberries, Cu concentrations in lingonberries, and Ni concentration in mushrooms (*Leccinum* genus) increased from the site closest to the smelter to the site 19 km away, and then decreased in sites beyond that. Cu, Cd, and Hg concentrations in mushrooms (*Leccinum* genus) at the furthest site were 176–209% of those at the closest site. 

Compared to the border regions of Norway and Russia, samples from the border region of Finland showed lower Ni and As concentrations in bilberries, and lower Ni, As, and Cd concentrations in lingonberries (Table S1). In total, 84% of samples from all border regions were above the maximum concentrations observed at the reference sites. Ni and As concentrations in bilberries; Ni, Cu, Co, As, Pb, and Cd concentrations in lingonberries; and Ni, Cu, As, Cd, and Hg concentrations in mushrooms (*Leccinum* genus) were higher in the border regions than at the reference sites. This was also the case for Co, Cd, and Pb concentrations in bilberries and Ni and As concentrations in cloudberries, but low concentrations at some sites in the border region resulted in overlapping ranges (Table S1).

Co and Pb concentrations in mushrooms (*Leccinum* genus) in the border regions were 0.095–1.2 and 0.16–0.93 times those at the reference site, respectively, and only one of seven mushroom samples from the border regions had Co concentrations above those observed at the reference site (Table S1).

#### 3.1.2. Spatial Trends in Fish Muscle Tissue

Ni, Co, and Cd concentrations in the pooled whitefish sample from the lake closest to the Ni-Cu smelter (Kuetsjarvi Lake, country, 2 km downstream from the city of Nikel) were 3.5 to 45 times those in whitefish samples from other lakes in Norway, Finland, and Russia (Table S1). Elevated Co concentrations were also observed in the single pike sample we had from Kuetsjarvi, with concentrations 2.7 to 8.0 times those observed in pike samples from other lakes. Moreover, Ni concentrations in pike samples from Russian lakes were 3.8 to 15 times those measured in pike samples from Norway and Finland, but no consistent trend was observed. We observed no trends in perch samples, although we had many samples from several lakes. 

#### 3.1.3. Spatial Trends in Game Muscle Tissue

The mean as concentration in single reindeer samples from Pasvik, country (approximately 20 km northwest of Nikel) in the Norwegian border region was 2.4 times that observed at Paistunturi (approximately 160 km northwest of Nikel) in Finland. Conversely, mean Pb and Cd concentrations in single reindeer samples from Paistunturi were 2.8 and 3.5 times those observed in Pasvik. 

Mean Ni, As, and Pb concentrations in moose samples from Pasvik were 2.4 to 11, 11 to 13, and 3.6 to 16 times those measured in single samples from Finland and Russia, respectively (Table S1). 

Hg concentrations were below LOD in all Norwegian reindeer and moose samples, and concentrations in moose from Russia and Finland were below LOD in Norwegian Hg analyses. Still, the Hg concentration in the single moose sample from Russia was 1.5 to 2.1 times the concentrations observed in single samples from Finland. 

### 3.2. Assessment of the Concentration of Toxic Elements in Food Groups

**Figure 5:**
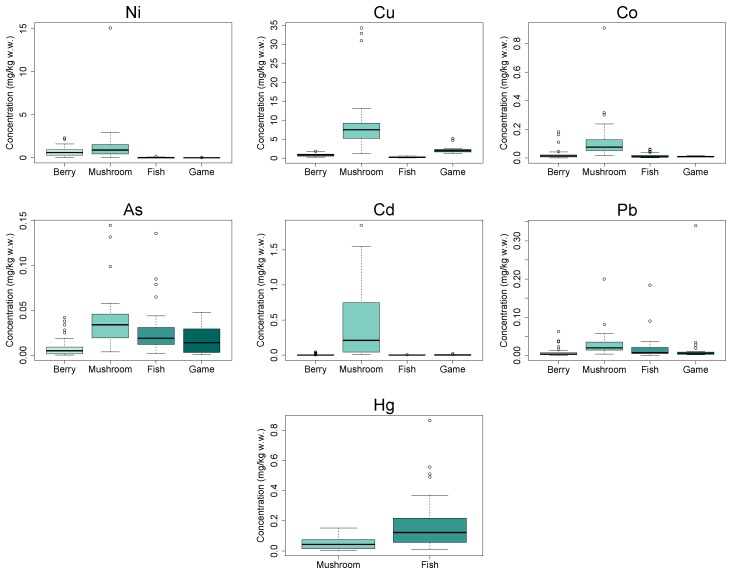
Ni, Cu, Co, As, Pb, Cd, and Hg concentrations in the four food groups. Berries and game were excluded from Hg analysis, since more than 60% of the concentrations were below limit of detection.

Independent of sampling site, mushrooms had higher Ni, Cu, Co, As, Pb, and Cd concentrations than berries, fish, and game (Figure 5). However, the difference in Ni concentrations between mushrooms and berries was not significant. Fish had significantly higher Hg concentrations than mushrooms, whereas more than 60% of berry and game samples were below LODs (these LODs were 1 to 3 orders of magnitude lower than the mean concentrations in fish and mushrooms). 

Concentrations varied between the species within each food group (Table S1). For example, Cd concentrations in cloudberries were up to three orders of magnitude higher than in the three other berry species, and the lowest concentration in cloudberries was 1.4 times the highest concentration observed in any of the three other berry species. However, for most toxic elements the concentration ranges overlapped between species within a food group. Variations between and within food groups were also observed in the principle component analyses (Figure S4a). 

Hg concentrations were highest in perch (Tjærebukta Lake, Norway) and pike (Rajakoski Lake, Russia) caught in lakes upstream of the city of Nikel in the Pasvik watercourse, 46 and 65 km from the smelter, respectively. 

All six samples of gypsy mushroom from Durvatn (30 km northeast of city of Nikel) and Gardsjøen (43 km northeast of the city of Nikel) (1.1–1.9 mg/kg w.w.) exceeded the European Union (EU) maximum levels for Cd concentrations in fungi (1.0 mg/kg w.w.). One of the two perch samples from Tjærebukta Lake (0.56 mg/kg w.w., 46 km upstream from the city of Nikel) and one of the five perch samples from Rundvatnet Lake (0.86 mg/kg w.w., 31 km north of the city of Nikel and not connected to the Pasvik watercourse) exceeded the EU maximum levels for Hg in fish muscle (0.50 mg/kg w.w.). One of the three moose samples from Pasvik (0.34 mg/kg w.w., approximately 20 km northwest of Nikel) exceeded the EU maximum level for Pb concentrations in meat (0.10 mg/kg w.w.) (Table S2).

## 4. Discussion

### 4.1. Spatial Trends in Local Wild Foods

In berries and mushrooms, all toxic elements we investigated, except Pb, Cd, and Hg concentrations in mushrooms (*Leccinum* genus), were highest at sites closest to the Ni-Cu smelter and generally decreased with increasing northeasterly distance. This decrease was most pronounced for Ni, As, and Co concentrations, which clearly indicates that the Ni-Cu smelter is a source of these toxic elements in berries and mushrooms. This same trend has previously been confirmed for Ni and as concentrations in bilberries and cloudberries in the area [14,27]. Unfortunately, the distribution of our sampling sites for cloudberries hampered the evaluation of spatial trends in this study. In addition, Ni and As concentrations were elevated in the border regions compared to the reference sites, and were lower in the border region of Finland compared to the other border regions. Downward concentration gradients in the direction of prevailing winds have also been observed for Ni concentrations in lingonberries, crowberries, and mushrooms of the *Leccinum* genus (*L. aurantiacum* and *L. scabrum*), but not in bilberries, located near the Severonickel combine, another smelter in the city of Monchegorsk on the central Kola Peninsula [31]. The Ni-Cu smelters on the Kola Peninsula use different technologies and process different ores, which can lead to the emission of different elements [7]; therefore, emission trends cannot be directly compared. Previous studies on sediments, surface soil, terrestrial moss, and vegetation in the area support the hypothesis that the Ni-Cu smelter in the city of Nikel is a source of Ni, Co, and as in the surrounding area [3,4,5,7,9].

Although Cu is one of the main pollutants from the Ni-Cu smelter [1,4,7,14], we did not observe a clear spatial trend. Cu concentrations in lingonberries and mushrooms (*Leccinum* genus) from the border regions were elevated compared to those observed in reference sites, but this was not the case in bilberries or cloudberries. The elevated Cu concentrations in some berry and mushroom species in the border regions compared to reference sites could indicate that the Ni-Cu smelter is a contributing source of Cu in local wild food, an indication that is supported by two previous studies on bilberries and cloudberries in the area, although the reported spatial trends were inconsistent [14,27].

Pb and Cd concentrations were generally highest close to the Ni-Cu smelter, but we observed no clear spatial trends. The Ni-Cu smelter has been identified as a source of these toxic elements, but other local anthropogenic and geogenic sources (e.g., increased population density and traffic, late ban on leaded gasoline, increased geogenic dust levels, and coal burning) are likely also important contributors [4,7]. Despite this, most of the Pb concentrations we observed were not higher in the border regions than at the reference sites.

We observed no trend for Hg concentrations in mushrooms (*Leccinum* genus) in the border regions; thus our results do not indicate that the Ni-Cu smelter is a significant source of this element in mushrooms and berries. Still, Hg concentrations in mushrooms (*Leccinum* genus) were elevated in the border regions compared to the reference site, which is in agreement with geographical trends in moss samples from northern Norway [32]. 

The Ni and Co concentrations observed in mushrooms (*Leccinum* genus) in this study were up to two orders of magnitude higher than those reported in birch boletes (*Leccinum scabrum*) from three unpolluted sites in Poland (assuming 90% moisture content) [33]. Likewise, Cu and Hg concentrations were higher in the present study, but within the same magnitude, whereas Cd and Pb concentrations were slightly lower [33]. 

The elevated Ni, Co, and Cd concentrations in whitefish and the elevated Co concentrations in pike that we observed in Kuetsjarvi Lake are in agreement with previous studies on Ni and Cd in different tissues of whitefish and perch [6,10,13], and suggest that the smelter is a source of these toxic elements. Indeed, Kuetsjarvi Lake receives wastewater from both the Ni-Cu smelter and the city of Nikel, and, due to their close vicinity, it is difficult to distinguish the proportion of toxic elements they emit individually. We observed no spatial trend for Hg concentrations in fish, which is in agreement with previous studies from this area [10,13]. Still, concentrations in sediments have indicated Hg emission from the Ni-Cu smelter in addition to other local anthropogenic activities and long-range transport [3,5].

The muscle tissue samples from reindeer and moose were too few for a detailed spatial assessment. Still, reindeer samples from Pasvik, Norway had higher As concentrations than the samples from Finland, and moose samples from Pasvik had higher As concentrations than those from Finland and Russia. Elevated As concentrations have been previously observed in reindeer from Pasvik [34]. Reindeer from Paistunturi, Finland, had higher Cd and Pb concentrations than reindeer from Pasvik, but the reason for this is unclear.

Although spatial trends varied between species and toxic elements, our results clearly demonstrate that the toxic elements emitted from the Ni-Cu smelter can be found in local wild foods in the border regions between Norway, Finland, and Russia. The impact of the Ni-Cu smelter was most obvious in berries and mushrooms and most pronounced for Ni, As, and Co. While inconsistencies exist across studies for some toxic elements, all toxic elements evaluated in this study have previously been related to emissions from this Ni-Cu smelter [1,3,4,5,6,7,10,13,14]. Based on our data, it was not feasible to evaluate emissions from the briquette facility in the city of Zapolyarny (approximately 25 km east of Nikel) and the Ni-Cu smelter in the city of Nikel individually, and the proportion of toxic elements originating from each is unknown. In addition, other local anthropogenic or geogenic sources, as well as long-range transport, likely contribute to the presence of toxic elements in local wild foods in these border regions [4,7,35]. 

### 4.2. Assessment of the Concentration of Toxic Elements in Food Groups

Mushrooms had the highest concentrations of all the toxic elements we investigated, except Hg. This is not surprising, since previous studies have confirmed that wild mushrooms can accumulate high concentrations of toxic elements, especially mushrooms in the vicinity of pollution sources like metallurgic complexes, highways, major cities, landfills, or sewage sludge [36]. However, in our study we found no significant difference between Ni concentrations in mushrooms and berries. The higher Hg concentrations we observed in fish compared to the other food groups was also expected, since Hg, mainly in the form of highly toxic methylmercury, is known to bioaccumulate and biomagnify in the food chain, especially in aquatic systems [37,38].

Berries and mushrooms were not rinsed prior to analysis, as rinsing could remove toxic elements on these foods. Toxic elements are also taken up by plant root systems and mycelium [9,36], this is for example demonstrated for Ni in dwarf shrubs [9]. However, the fraction of toxic elements transferred into berries in this manner is unknown. 

### 4.3. Considerations of Risk by Wild Food Consumptions

Since the EU has not defined maximum permissible levels for toxic elements in foods that grow wild, we referred to values for comparable commercial species, which are set to protect public health [39,40,41,42]. The majority of samples were below the relevant EU maximum levels (Table S2), but six out of 12 samples of gypsy mushroom exceeded the EU maximum level for Cd; two out of 16 samples of perch exceeded the EU maximum level for Hg, and one out of 11 moose samples exceeded the EU maximum level for Pb. All these samples were from the Norwegian border region. Apart from these samples, the observed concentrations in the border regions and at reference sites would generally be considered safe for human consumption. However, the lack of applicable thresholds for many of the toxic elements we investigated makes it difficult to draw firm conclusions. This is exemplified by the highly elevated Cu and Ni concentrations we observed in a number of mushroom samples in Russia and Norway; these samples had up to four times (Cu) and 10 times (Ni) the mean values for all mushrooms. No association could be established between high Ni and Cu concentrations and mushroom species or geographical locations. The elevated Ni and Cu concentrations observed in mushrooms could pose a risk to people consuming large amounts on a regular basis. In such cases, the composition of species consumed, as well as the harvesting areas, must be taken into account when evaluating safety for consumption. 

To further evaluate the risk associated with consumption of wild food, we employed the concepts tolerable weekly intake (TWI) and daily intake (TDI). We estimated how much of a food can safely be consumed based on the observed element concentrations. Of the elements that exceeded the EU maximum levels, TWIs are available for Cd (2.5 μg/kg body weight (b.w.)) and Hg (4 and 1.3 μg/kg b.w. for Hg and methyl-Hg, respectively) [43,44]. A TDI has also been set for Ni (2.8 μg/kg b.w.) [45]. Based on the samples with the highest concentrations, a person weighing 70 kg can on a yearly basis eat 5.5 kg of perch from Rundvannet, Norway before the TWI for methyl-Hg is reached, assuming that all Hg in the fish sample was methyl-Hg (a high proportion of methyl-Hg was frequently reported to the European Food Safety Authority) [44]. Further, 4.8 kg of gypsy mushroom from Durvatn, Norway can be eaten on a yearly basis, before the TWI for Cd is reached, and 4.7 kg of rollrim milkcap can be eaten from the Nikel site in Russia before the TDI for Ni is reached. It should be noted that we have not considered intake of elements from other sources in these calculations, but most local people will never reach these intake values. The risk assessments are likewise limited by our lack of food intake information from the local populations. Still, this implies that moderate consumption of wild food from the study area, except close to the smelter, could be considered safe. For additional certainty, however, the individual’s dietary habits should be assessed in greater detail.

When considering the actual risk posed by the toxic elements measured in wild food it is important to recognize that most people consume considerably less wild food than commercial food. Unquestionably, some individuals do rely heavily on local wild food, but considering that we lack detailed intake data, this issue is not discussed further.

The local wild foods examined in this study have low fat contents and contain many important nutrients, such as vitamins, minerals, and fatty acids [46]. Furthermore, the activities of fishing, hunting, and picking berries and mushrooms are of cultural importance and can increase outdoor activity. In this way, the use of local wild foods can contribute to a healthy lifestyle, and concerns about contamination might discourage the use of these important and readily available resources.

### 4.4. Limitations of the Study

The evaluation of spatial trends was limited by the distribution of sampling sites for several species. However, sampling sites were chosen based on how frequently they are used for collecting local wild food, and thus represent important sites in relation to human exposure. Sampling sites were mainly located northeast or southwest of the Ni-Cu smelter, which made it difficult to identify spatial patterns in other directions. 

Several of our fish and game samples came from individual specimens. This is important to take into account when comparing with results from pooled samples, since samples from individual animals are likely not representative of the entire population in the area. Because of the small number of game samples in this study, and the fact that wild game can move over large areas, it is difficult to determine the exact area each game sample represents. However, the Norwegian and Finnish reindeer move within their grazing district, which is a defined area with specific summer and winter grazing areas. In addition, the size and age of the animals, which can influence element levels, were not evaluated [47,48,49]. 

Although the different laboratories used different reference materials, all three laboratories that participated in this study adhere to a quality assurance/control program, thus ensuring the general comparability of laboratory results. However, the LOD values for some toxic elements varied across the laboratories, making spatial trend assessment difficult in some cases. 

## 5. Conclusions

To our knowledge this is the most extensive study on toxic elements in local wild foods from the border regions between Norway, Finland, and Russia. Our results demonstrate that toxic elements emitted from the Ni-Cu smelter are found in local wild food, especially in Russia, where the Ni-Cu smelter is located, but also in areas of Norway, mainly northeast of the smelter, and to a lesser extent in Finland. The impact of the smelter was reflected most strongly in the observed Ni, Co, and as concentrations and was most obvious in berries and mushrooms. The suggestion that the Ni-Cu smelter is the source of Ni and Co emissions was supported by the elevated concentrations of these toxic elements in fish from Kuetsjarvi Lake, located close to the smelter. 

Among the four food groups we investigated, mushrooms had higher Ni, Cu, Co, As, Pb, and Cd concentrations than berries, fish, and game, whereas fish had the highest Hg concentrations. The absolute majority of samples had concentrations below EU maximum levels for comparable commercial foods and can be considered safe for human consumption. However, the elevated Ni and Cu concentrations observed in some mushroom samples could pose a risk to people who frequently consume mushrooms. These people should avoid collecting mushrooms close to the smelter and in other areas that showed high concentrations of toxic elements in mushroom species. 

This study confirms that toxic elements emitted from the smelter are found in local wild food, but concentrations are highly dependent on area, food type, and toxic element.

## Figures and Tables

**Table 1:** Number of sampling sites and samples collected according to food groups and species in Norway, Russia, and Finland.

	Norway *		Russia		Finland	
	*n* Sites	*n* Samples	*n* Sites	*n* Samples	*n* Sites	*n* Samples
**Berries**	**16**	**56**	**4**	**10**	**3**	**9**
Bilberry (*Vaccínium myrtíllus*)	8	20	4	4	1	3
Lingonberry (*Vaccínium vítis-idaéa*)	6	16	4	4	2	3
Cloudberry (*Rubus chamaemorus*)	7	11	-	-	1	3
Crowberry (*Empetrum nigrum*)	3	9	1	1	-	-
Bog bilberry (*Vaccínium uliginósum*)	-	-	1	1	-	-
**Mushrooms**	**6**	**24**	**4**	**8**	**2**	**3**
Orange birch bolete (*Leccinum versipelle*)	4	12	-	-	-	-
Orange oak bolete (*Leccinum aurantiacum*)	-	-	4	4	-	-
Birch bolete (*Leccinum scabrum*)	-	-	1	1	-	-
Gypsy mushroom (*Cortinarius caperatus*)	4	12	-	-	-	-
Rufous milkcap (*Lactarius rufus*)	-	-	-	-	1	1
Bearded milkcap (*Lactarius torminosus*)	-	-	1	1	-	-
Rollrim milkcap (*Lactarius resimus*)	-	-	1	1	-	-
Russula (*Russulaceae*)	-	-	1	1	-	-
Mixed mushrooms	-	-	-	-	1	2
**Fish muscle**	7 (lakes) **	**19**	5 (lakes) **	**14**	2 (lakes) **	**8**
Whitefish (*Coregonus lavaretus*)	2	2	4	4	1	3
Perch (*Perca fluviatilis*)	3	8	4	4	2	4
Pike (*Esox lucius*)	2	2	4	4	1	1
Arctic char (*Salvelinus alpinus*)	3	3	1	1	-	-
Brown trout (*Salmo trutta*)	4	4	1	1	-	-
**Game muscle**	2 (region) **	**13**	1 (region) **	**1**	6 (region) **	**9**
Reindeer (*Rangifer tarandus*)	1	10	-	-	1	3
Moose (*Alces alces*)	1	3	1	1	3	3
Ptarmigan (*Lagopus lagopus*)	-	-	-	-	2	3

*n*: number of sites or samples, * The Norwegian numbers include the reference sites in Norway (see Table S1 for details). ** Regions or lakes where the animals move within a relatively large area.

## Supplementary Materials

The following are available online at www.mdpi.com/1660-4601/14/7/694/s1, Figure S1: Concentration (mg/kg w.w.) of toxic elements in lingonberries, Figure S2: Concentration (mg/kg w.w.) of toxic elements in crowberries, Figure S3: Concentration of toxic elements in mushrooms (*Leccinum* genus), Figure S4: Principal component biplots, Table S1: Concentration (mg/kg w.w.) of toxic elements in berries, mushrooms, fish, reindeer, and moose, Table S2: European Union maximum levels for toxic elements and food items compared with results from the present study.
