# Supplementary material for: The Impact of a Nickel-Copper Smelter on Concentrations of Toxic Elements in Local Wild Food from the Norwegian, Finnish, and Russian Border Regions
Source: Int J Environ Res Public Health. 2017 Jun 28;14(7):694. doi: 10.3390/ijerph14070694 (PMC5551132; doi:10.3390/ijerph14070694)
Supplement: Supplementary file 1 [file ijerph-14-00694-s001.pdf]

Supplementary:

Table S1. Concentration (mg/kg w.w.) of toxic elements in berries, mushrooms, fish, reindeer, and moose from the different sampling sites

	Coordinates (Lat;Long)	Ni	Cu	Co	As	Cd	Pb	Hg
**Berries**								
**Bilberries**								
Jordanfoss (Norway)	69.2093;29.29568							
no. of pools		3	3	3	3	3	3	3
mean conc.		0.10	0.41	0.0012	0.0014	0.000097	0.0031	< 0.005
median conc.		0.10	0.42	0.0012	0.0014	0.00014	0.0028	
min;max conc.		0.077;0.13	0.30;0.52	0.00080;.0016	0.00094;0.0019	0;.00015	0.0024;0.0040	
n < LOD		-	-	-	-	-	-	3
Durvatn (Norway)	69.61505;30.70556							
no. of pools		3	3	3	3	3	3	3
mean conc.		0.72	0.71	0.0094	0.0077	0.0011	0.0033	< 0.005
median conc.		0.70	0.78	0.0087	0.0074	0.00069	0.0023	
min;max conc.		0.61;0.83	0.50;0.85	0.0075;0.012	0.0054;0.010	0.00068;0.0018	0.0023;0.0053	
n < LOD		-	-	-	-	-	-	3
Gardsjøen (Norway)	69.71014;30.86115							
n of pools		3	3	3	3	3	3	3
mean conc.		0.32	0.55	0.0052	0.0040	0.00072	0.0023	< 0.005
median conc.		0.31	0.53	0.0053	0.0042	0.00068	0.0021	
min;max conc.		0.31;0.36	0.52;0.61	0.0048;0.0054	0.0032;0.0046	0.00067;0.00082	0.0020;0.0029	
n < LOD		-	-	-	-	-	-	3
Holmvatn (Norway)	69.71202;30.75161							
no. of pools		3	3	3	3	3	3	3
mean conc.		0.32	0.57	0.0060	0.0042	0.00052	0.0030	< 0.005
median conc.		0.32	0.083	0.0059	0.0046	0.00037	0.0033	
min;max conc.		0.31;0.33	0.50;0.66	0.0058;0.0064	0.0031;0.0047	0.00026;0.00092	0.0025;0.0033	
n < LOD		-	-	-	-	-	-	3

Table S1. *Cont.*

	Coordinates (Lat;Long)	Ni	Cu	Co	As	Cd	Pb	Hg
Grensa (Norway)<br>no. of pools<br>mean conc.<br>median conc.<br>min;max conc.<br>n < LOD	69.56039;30.93146	 3<br>0.73<br>0.75<br>0.65;0.79<br>-	 3<br>0.44<br>0.38<br>0.37;0.57<br>-	 3<br>0.011<br>0.012<br>0.011;0.012<br>-	 3<br>0.0049<br>0.0047<br>0.0035;0.0064<br>-	 3<br>0.000076<br>0.00077<br>0.00073;0.00077<br>-	 3<br>0.00044<br>0.00024<br>0;0.0011<br>-	 3<br>< 0.005<br><br><br>3
Børsevatn (Norway)<br>no. of pools<br>mean conc.<br>median conc.<br>min;max conc.<br>n < LOD	69.55638;30.58504	 3<br>0.93<br>0.82<br>0.75;1.22<br>-	 3<br>1.0<br>0.79<br>0.73;1.58<br>-	 3<br>0.019<br>0.018<br>0.013;0.027<br>-	 3<br>0.022<br>0.017<br>0.014;0.034<br>-	 3<br>0.0021<br>0.0011<br>0.00090;0.0042<br>-	 3<br>0.012<br>0.0077<br>0.0058;0.023<br>-	 3<br>< 0.005<br><br><br>3
Tromsøya, ref. (Norway)<br>no. of pools<br>Sample conc.<br>n < LOD	69.68977;18.96587	 1<br>0.030<br>-	 1<br>0.43<br>-	 1<br>0.0020<br>-	 1<br>0.00020<br>-	 1<br>0.00014<br>-	 1<br>0.0020<br>-	 -
Stålvikbotn Balsfjord, ref. (Norway)<br>no. of pools<br>Sample conc.<br>n < LOD	69.30667;18.86819	 <br>1<br>0.04<br>-	 <br>1<br>0.56<br>-	 <br>1<br>0.0010<br>-	 <br>1<br>0.00030<br>-	 <br>1<br>0.00030<br>-	 <br>1<br>0.0020<br>-	 <br>-
Nikel site (Russia)<br>no. of pools<br>Sample conc.<br>n < LOD	69.44544;30.49665	 1<br>1.1<br>-	 1<br>0.86<br>-	 1<br>0.038<br>-	 1<br>0.019<br>-	 1<br>0.001<br>-	 1<br>0.013<br>-	 1<br>< 0.0005<br>1
North (Russia)<br>no. of pools<br>Sample conc.<br>n < LOD	69.54455;30.92999	 1<br>0.60<br>-	 1<br>0.32<br>-	 1<br>0.014<br>-	 1<br>0.0030<br>-	 1<br>< 0.0005<br>1	 1<br>< 0.005<br>1	 1<br>< 0.0005<br>1

Table S1. *Cont.*

	**Coordinates (Lat;Long)**	**Ni**	**Cu**	**Co**	**As**	**Cd**	**Pb**	**Hg**
East (Russia)<br>no. of pools<br>Sample conc.<br>n < LOD	69.37252;30.96479	 1<br>0.50<br>-	 1<br>0.47<br>-	 1<br>0.013<br>-	 1<br>0.0020<br>-	 1<br>< 0.0005<br>1	 1<br>0.0060<br>-	 1<br>0.0090<br>-
South (Russia)<br>n of pools<br>Sample conc.<br>n < LOD	69.22005;30.15659	 1<br>0.50<br>-	 1<br>0.50<br>-	 1<br>0.012<br>-	 1<br>0.0010<br>-	 1<br>0.0010<br>-	 1<br>0.0060<br>-	 1<br>< 0.0005<br>1
Nellim (Finland)<br>n of pools<br>mean conc.<br>median conc.<br>min;max conc.<br>n < LOD	68.86748;28.39533	 3<br>0.10<br>0.11<br>0.081;0.13<br>-	 3<br>0.81<br>0.83<br>0.76;0.83<br>-	 -	 3<br>0.0010<br>0.00070<br>0.00060;0.0017<br>-	 3<br>0.00059<br>0.00067<br>0.00039;0.00071<br>-	 3<br><br>< 0.005<br><br>3	 3<br>0.00086<br>0.00090<br>0.00053;0.00116<br>-
**Lingonberries**								
Durvatn (Norway)<br>n of pools<br>mean conc.<br>median conc.<br>min;max conc.<br>n < LOD	69.61505;30.70556	 3<br>0.87<br>0.90<br>0.80;0.92<br>-	 3<br>0.97<br>0.90<br>0.88;1.1<br>-	 3<br>0.018<br>0.018<br>0.016;0.020<br>-	 3<br>0.0093<br>0.0093<br>0.0091;0.0096<br>-	 3<br>0.00065<br>0.00070<br>0.00056;0.00070<br>-	 3<br>0.0066<br>0.0071<br>0.0049;0.0080<br>-	 3<br>< 0.005<br><br><br>3
Gardsjøen (Norway)<br>n of pools<br>mean conc.<br>median conc.<br>min;max conc.<br>n < LOD	69.71014;30.86115	 3<br>0.73<br>0.72<br>0.66;0.80<br>-	 3<br>0.81<br>0.78<br>0.73;0.93<br>-	 3<br>0.018<br>0.018<br>0.016;0.020<br>-	 3<br>0.0068<br>0.0067<br>0.0057;0.0079<br>-	 3<br>0.00051<br>0.00054<br>0.00037;0.00061<br>-	 3<br>0.0096<br>0.0091<br>0.0082;0.011<br>-	 3<br>< 0.005<br><br><br>3

Table S1.*Cont.*

	**Coordinates (Lat;Long)**	**Ni**	**Cu**	**Co**	**As**	**Cd**	**Pb**	**Hg**
Grensa (Norway)<br>n of pools<br>mean conc.<br>median conc.<br>min;max conc.<br>n < LOD	69.56039;30.93146	 3<br>1.0<br>1.0<br>0.95;1.2<br>-	 3<br>0.71<br>0.73<br>0.63;0.76<br>-	 3<br>0.025<br>0.024<br>0.022;0.029<br>-	 3<br>0.0088<br>0.0085<br>0.0083;0.0097<br>-	 3<br>0.00043<br>0.00044<br>0.00039;0.00047<br>-	 3<br>0.0029<br>0.0027<br>0.0022;0.0037<br>-	 3<br>< 0.005<br><br><br>3
Børsevatn (Norway)<br>n of pools<br>mean conc.<br>median conc.<br>min;max conc.<br>n < LOD	69.55638;30.58504	 3<br>1.4<br>1.5<br>1.3;1.5<br>-	 3<br>1.2<br>1.1<br>1.1;1.3<br>-	 3<br>0.038<br>0.038<br>0.034;0.041<br>-	 3<br>0.027<br>0.028<br>0.025;0.028<br>-	 3<br>0.00077<br>0.00082<br>0.00062;0.00088<br>-	 3<br>0.014<br>0.013<br>0.012;0.016<br>-	 3<br>< 0.005<br><br><br>3
Sollia Jarfjord (Norway)<br>n of pools<br>mean conc.<br>median conc.<br>min;max conc.<br>n < LOD	69.66485;30.19825	 3<br>0.51<br>0.47<br>0.37;0.70<br>-	 3<br>0.68<br>0.67<br>0.56;0.81<br>-	 3<br>0.0096<br>0.0086<br>0.0060;0.014<br>-	 3<br>0.0067<br>0.0063<br>0.0054;0.0083<br>-	 3<br>0.00058<br>0.00053<br>0.00046;0.00075<br>-	 3<br>0.0042<br>0.0038<br>0.0032;0.0058<br>-	 3<br>< 0.005<br><br><br>3
Stålvikbotn Balsfjord, ref. (Norway)<br>n of pools<br>Sample conc.<br>n < LOD	69.30667;18.86819	 <br>1<br>0.030<br>-	 <br>1<br>0.43<br>-	 <br>1<br>0.0020<br>-	 <br>1<br>0.00014<br>-	 <br>1<br>0.00014<br>-	 <br>1<br>0.0020<br>-	 <br>-
Nikel site (Russia)<br>n of pools<br>Sample conc.<br>n < LOD	69.44544;30.49665	 1<br>2.3<br>-	 1<br>0.96<br>-	 1<br>0.16<br>-	 1<br>0.042<br>-	 1<br>0.0010<br>-	 1<br>0.036<br>-	 1<br>< 0.0005<br>1

Table S1.*Cont.*

	Coordinates (Lat;Long)	Ni	Cu	Co	As	Cd	Pb	Hg
South (Russia)<br>n of pools<br>Sample conc.<br>n < LOD	69.22005;30.15659	 1<br>0.70<br>-	 1<br>0.60<br>-	 1<br>0.017<br>-	 1<br>0.0070<br>-	 1<br>< 0.0005<br>1	 1<br>0.038<br>-	 1<br>< 0.0005<br>1
East (Russia)<br>n of pools<br>Sample conc.<br>n < LOD	69.37252;30.96479	 1<br>1.2<br>-	 1<br>0.92<br>-	 1<br>0.045<br>-	 1<br>0.010<br>-	 1<br>0.0010<br>-	 1<br>0.013<br>-	 1<br>< 0.0005<br>1
North (Russia)<br>n of pools<br>Sample conc.<br>n < LOD	69.54455;30.92999	 1<br>1.3<br>-	 1<br>0.84<br>-	 1<br>0.11<br>-	 1<br>0.014<br>-	 1<br>0.0010<br>-	 1<br>0.0070<br>-	 1<br>< 0.0005<br>1
Inari (Finland)<br>n of pools<br>Sample conc.<br>n < LOD	68.82956;26.6129	 1<br>0.071<br>-	 1<br>0.84<br>-	 -	 1<br>0.00080<br>-	 1<br>< 0.00025<br>1	 1<br>< 0.005<br>1	 1<br>0.00026<br>-
Nellim (Finland)<br>n of pools<br>mean conc.<br>median conc.<br>min;max conc.<br>n < LOD	68.848;28.30347	 2<br>0.096<br>0.096<br>0.089;0.10<br>-	 2<br>0.84<br>0.84<br>0.83;0.85<br>-	 -	 2<br>0.0013<br>0.0013<br>0.0012;0.0013<br>-	 2<br>0.00033<br>0.00033<br>0.00026;0.00040<br>-	 2<br>< 0.005<br><br><br>2	 2<br>0.00049<br>0.00049<br>0.00041;0.00056<br>-
**Cloudberries**								
Jarfjord (Norway)<br>n of pools<br>mean conc.<br>median conc.<br>min;max conc.<br>n < LOD	69.55738;30.84469	 3<br>0.51<br>0.49<br>0.45;0.57<br>-	 3<br>1.2<br>1.1<br>1.1;1.4<br>-	 3<br>0.012<br>0.012<br>0.011;0.013<br>-	 3<br>0.0026<br>0.0025<br>0.0024;0.0029<br>-	 3<br>0.032<br>0.029<br>0.024;0.043<br>-	 3<br>0.0017<br>0.0013<br>0.00082;0.0030<br>-	 3<br>< 0.005<br><br><br>3

Table S1. *Cont.*

	Coordinates (Lat;Long)	Ni	Cu	Co	As	Cd	Pb	Hg
Pasvik (Norway)<br>n of pools<br>mean conc.<br>median conc.<br>min;max conc.<br>n < LOD	69.37656;29.68486	 3<br>1.4<br>1.3<br>1.2;1.6<br>-	 3<br>1.4<br>1.6<br>1.1;1.6<br>-	 3<br>0.013<br>0.012<br>0.012;0.014<br>-	 3<br>0.0043<br>0.0043<br>0.0036;0.0051<br>-	 3<br>0.017<br>0.017<br>0.014;0.019<br>-	 3<br>0.0017<br>0.0015<br>0.0012;0.0023<br>-	 3<br>< 0.005<br><br><br>3
Pasvik Skogfoss (Norway)<br>n of pools<br>Sample conc.<br>n < LOD	69.3692;29.68718	1<br>0.59<br>-	1<br>0.53<br>-	1<br>0.019<br>-	1<br>0.0023<br>-	1<br>0.0064<br>-	1<br>0.0040<br>-	-
Øvre Pasvik (Norway)<br>n of pools<br>Sample conc.<br>n < LOD	69.08881;28.94608	1<br>0.17<br>-	1<br>0.59<br>-	1<br>0.0030<br>-	1<br>0.00020<br>-	1<br>0.0059<br>-	1<br>0.002<br>-	-
Kjelmøya (Norway)<br>n of pools<br>Sample conc.<br>n < LOD	69.86874;30.0554	1<br>0.60<br>-	1<br>1.7<br>-	1<br>0.012<br>-	1<br>0.0012<br>-	1<br>0.040<br>-	1<br>0.0060<br>-	-
Lakseidet Balsfjord, ref. (Norway)<br>n of pools<br>Sample conc.<br>n < LOD	69.29649;18.95063	1<br>0.23<br>-	1<br>1.3<br>-	1<br>0.010<br>-	1<br>0.00030<br>-	1<br>0.012<br>-	1<br>0.0020<br>-	-
Storsteines, ref. (Norway)<br>n of pools<br>Sample conc.<br>n < LOD	69.24052;19.23586	1<br>0.12<br>-	1<br>1.0<br>-	1<br>0.0080<br>-	1<br>0.00020<br>-	1<br>0.0098<br>-	1<br>0.0010<br>-	-

Table S1. *Cont.*

	Coordinates (Lat;Long)	Ni	Cu	Co	As	Cd	Pb	Hg
Nellim (Finland)	68.86748;28.39533							
n of pools		3	3	-	3	3	3	3
mean conc.		0.36	1.2		0.0019	0.021		0.00073
median conc.		0.34	1.4		0.0014	0.021		0.00078
min;max conc.		0.31;0.44	0.83;1.5		0.0010;0.0033	0.013;0.028	<0.005;0.0084	0.00045;0.00096
n < LOD		-	-		-	-	2	-
**Crowberries**								
Jordanfoss (Norway)	69.2093;29.29568							
n of pools		3	3	3	3	3	3	3
mean conc.		0.14	0.62	0.0012	0.0013	0.00024	0.0032	< 0.005
median conc.		0.13	0.46	0.0017	0.0012	0.00011	0.0026	
min;max conc.		0.11;0.18	0.43;0.97	0.000038;0.0018	0.00090;0.0018	0.00011;0.00049	0.00088;0.0061	
n < LOD		-	-	-	-	-	-	3
Durvatn (Norway)	69.61505;30.70556							
n of pools		3	3	3	3	3	3	3
mean conc.		0.81	1.3	0.020	0.0095	0.00054	0.0088	< 0.005
median conc.		0.83	1.1	0.020	0.011	0.00057	0.0093	
min;max conc.		0.64;0.96	0.91;1.8	0.015;0.024	0.0069;0.011	0.00046;0.00061	0.0057;0.011	
n < LOD		-	-	-	-	-	-	3
Grensa (Norway)	69.56039;30.93146							
n of pools		3	3	3	3	3	3	3
mean conc.		1.1	1.1	0.025	0.0073	0.00048	0.0059	< 0.005
median conc.		1.2	1.0	0.026	0.0068	0.00050	0.0064	
min;max conc.		0.98;1.2	0.95;1.4	0.022;0.028	0.0065;0.0085	0.00043;0.00051	0.0047;0.0067	
n < LOD		-	-	-	-	-	-	3
Nikel site (Russia)	69.44544;30.49665							
n of pools		1	1	1	1	1	1	1
Sample conc.		2.2	1.6	0.18	0.038	0.0020	0.063	0.0010
n < LOD		-	-	-	-	-	-	-

Table S1. *Cont.*

	Coordinates (Lat;Long)	Ni	Cu	Co	As	Cd	Pb	Hg
**Bog bilberry**								
Nikel site (Russia)	69.44544;30.49665							
n of pools		1	1	1	1	1	1	1
Sample conc.		0.78	0.44	0.018	0.017	0.0078	0.0094	< 0.0005
n < LOD		-	-	-	-	-	-	1
**Mushrooms**								
**Orange birch bolete**								
Durvatn (Norway)	69.61505;30.70556							
n of pools		3	3	3	3	3	3	3
mean conc.		0.37	7.6	0.018	0.031	0.40	0.018	0.077
median conc.		0.38	7.5	0.018	0.032	0.40	0.018	0.076
min;max conc.		0.33;0.41	7.1;8.2	0.016;0.020	0.028;0.034	0.32;0.49	0.016;0.019	0.069;0.088
n < LOD		-	-	-	-	-	-	-
Grensa (Norway)	69.56039;30.93146							
n of pools		3	3	3	3	3	3	3
mean conc.		0.75	8.8	0.041	0.049	0.20	0.014	0.083
median conc.		0.74	8.7	0.040	0.051	0.18	0.014	0.076
min;max conc.		0.71;0.80	8.0;9.8	0.036;0.047	0.044;0.052	0.18;0.24	0.013;0.014	0.071;0.10
n < LOD		-	-	-	-	-	-	-
Børsevatn (Norway)	69.55638;30.58504							
n of pools		3	3	3	3	3	3	3
mean conc.		1.5	11.5	0.090	0.050	0.040	0.037	0.15
median conc.		1.4	11.1	0.088	0.046	0.038	0.037	0.15
min;max conc.		1.3;1.7	10.3;13.1	0.079;0.10	0.046;0.058	0.036;0.047	0.029;0.045	0.13;0.15
n < LOD		-	-	-	-	-	-	-
Tønsvika, ref. (Norway)	69.74198;19.17172							
n of pools		3	3	3	3	3	3	3
mean conc.		0.28	3.4	0.19	0.025	0.022	0.045	< 0.005
median conc.		0.24	3.8	0.17	0.020	0.020	0.040	
min;max conc.		0.11;0.50	2.5;3.8	0.099;0.30	0.017;0.036	0.015;0.031	0.014;0.081	
n < LOD		-	-	-	-	-	-	3

Table S1. *Cont.*

	Coordinates (Lat;Long)	Ni	Cu	Co	As	Cd	Pb	Hg
**Orange oak bolete**								
Nikel site (Russia)<br>n of pools<br>Sample conc.<br>n < LOD	69.44544;30.49665	 1<br>1.4<br>-	 1<br>6.2<br>-	 1<br>0.24<br>-	 1<br>0.15<br>-	 1<br>0.21<br>-	 1<br>0.032<br>-	 1<br>0.051<br>-
South (Russia)<br>n of pools<br>Sample conc.<br>n < LOD	69.22005;30.15659	 1<br>0.32<br>-	 1<br>4.9<br>-	 1<br>0.043<br>-	 1<br>0.028<br>-	 1<br>0.14<br>-	 1<br>0.040<br>-	 1<br>0.14<br>-
East (Russia)<br>n of pools<br>Sample conc.<br>n < LOD	69.37252;30.96479	 1<br>0.63<br>-	 1<br>7.15<br>-	 1<br>0.063<br>-	 1<br>0.050<br>-	 1<br>0.30<br>-	 1<br>0.042<br>-	 1<br>0.086<br>-
North (Russia)<br>n of pools<br>Sample conc.<br>< LOD	69.54455;30.92999	 1<br>0.91<br>-	 1<br>7.1<br>-	 1<br>0.070<br>-	 1<br>0.040<br>-	 1<br>0.21<br>-	 1<br>0.0070<br>-	 1<br>0.12<br>-
**Birch bolete**								
Nikel site (Russia)<br>n of pools<br>Sample conc.<br>n < LOD	69.44544;30.49665	 1<br>1.4<br>-	 1<br>2.4<br>-	 1<br>0.22<br>-	 1<br>0.099<br>-	 1<br>0.18<br>-	 1<br>0.043<br>-	 1<br>0.023<br>-
**Gypsy mushroom**								
Durvatn (Norway)<br>n of pools<br>mean conc.<br>median conc.<br>min;max conc.<br>n < LOD	69.61505;30.70556	 3<br>1.0<br>1.0<br>0.96;1.1<br>-	 3<br>9.9<br>10.3<br>8.6;10.9<br>-	 3<br>0.062<br>0.063<br>0.054;0.069<br>-	 3<br>0.047<br>0.046<br>0.043;0.052<br>-	 3<br>1.6<br>1.5<br>1.3;1.9<br>-	 3<br>0.012<br>0.011<br>0.010;0.015<br>-	 3<br>0.017<br>0.017<br>0.013;0.021<br>-

Table S1. *Cont.*

	Coordinates (Lat;Long)	Ni	Cu	Co	As	Cd	Pb	Hg
Gardsjøen (Norway)<br>n of pools<br>mean conc.<br>median conc.<br>min;max conc.<br>n < LOD	69.71014;30.86115	 3<br>0.79<br>0.78<br>0.69;0.90<br>-	 3<br>7.7<br>7.5<br>7.5;8.1<br>-	 3<br>0.13<br>0.13<br>0.12;0.14<br>-	 3<br>0.015<br>0.015<br>0.014;0.016<br>-	 3<br>1.3<br>1.2<br>1.1;1.5<br>-	 3<br>0.019<br>0.019<br>0.019;0.020<br>-	 3<br>0.047<br>0.045<br>0.044;0.053<br>-
Grensa (Norway)<br>n of pools<br>mean conc.<br>median conc.<br>min;max conc.<br>n < LOD	69.56039;30.93146	 3<br>2.8<br>2.8<br>2.8;2.9<br>-	 3<br>32.7<br>32.9<br>30.9;34.3<br>-	 3<br>0.12<br>0.12<br>0.12;0.12<br>-	 3<br>0.034<br>0.035<br>0.032;0.035<br>-	 3<br>0.91<br>0.94<br>0.81;1.0<br>-	 3<br>0.022<br>0.021<br>0.021;0.025<br>-	 3<br>0.046<br>0.047<br>0.039;0.053<br>-
Sollia Jarfjord<br>n of pools<br>mean conc.<br>median conc.<br>min;max conc.<br>n < LOD	69.66485;30.19825	 3<br>1.8<br>1.8<br>1.7;1.9<br>-	 3<br>6.1<br>5.8<br>5.8;6.6<br>-	 3<br>0.066<br>0.068<br>0.058;0.071<br>-	 3<br>0.024<br>0.024<br>0.024;0.024<br>-	 3<br>0.57<br>0.54<br>0.49;0.68<br>-	 3<br>0.013<br>0.012<br>0.011;0.014<br>-	 3<br>0.020<br>0.020<br>0.017;0.022<br>-
**Bearded milkcap**								
South (Russia)<br>n of pools<br>Sample conc.<br>n < LOD	69.22005;30.15659	 1<br>0.69<br>-	 1<br>1.2<br>-	 1<br>0.049<br>-	 1<br>0.019<br>-	 1<br>0.0086<br>-	 1<br>0.021<br>-	 1<br>0.0037<br>-
**Rollrim milkcap**								
Nikel site (Russia)<br>n of pools<br>Sample conc.<br>n < LOD	69.44544;30.49665	 1<br>15.1<br>-	 1<br>8.6<br>-	 1<br>0.91<br>-	 1<br>0.13<br>-	 1<br>0.15<br>-	 1<br>0.20<br>-	 1<br>0.014<br>-

Table S1.*Cont.*

	Coordinates (Lat;Long)	Ni	Cu	Co	As	Cd	Pb	Hg
**Russula**								
North (Russia)<br>n of pools<br>Sample conc.<br>< LOD	69.54455;30.92999	 1<br>2.4<br>-	 1<br>3.7<br>-	 1<br>0.32<br>-	 1<br>0.014<br>-	 1<br>0.12<br>-	 1<br>0.033<br>-	 1<br>0.0037<br>-
**Rufous milkcap**								
Inari (Finland)<br>n of pools<br>Sample conc.<br>n < LOD	68.82956;26.6129	 1<br>0.083<br>-	 1<br>2.3<br>-	 -	 1<br>0.0038<br>-	 1<br>0.032<br>-	 1<br>0.057<br>-	 1<br>0.0032<br>-
**Mixed mushrooms**								
Nellim (Finland)<br>n of pools<br>mean conc.<br>median conc.<br>min;max conc.<br>n < LOD	68.848;28.30347	 2<br>0.061<br>0.061<br>0.037;0.085<br>-	 2<br>3.7<br>3.7<br>1.8;5.6<br>-	 -	 2<br>0.014<br>0.014<br>0.013;0.016<br>-	 2<br>0.032<br>0.032<br>0.023;0.042<br>-	 2<br>0.017<br>0.017<br>0.0035;0.031<br>-	 2<br>0.014<br>0.014<br>0.013;0.015<br>-
**Fish**								
**Whitefish**								
Tjærebukta (Norway)<br>n of pools<br>n of single samples<br>Sample conc.<br>n < LOD	69.22725;29.18445	 -<br>1<br>0.0070<br>-	 -<br>1<br>0.23<br>-	 -<br>1<br>0.011<br>-	 -<br>1<br>0.085<br>-	 -<br>1<br>0.00029<br>-	 -<br>1<br>0.0058<br>-	 -<br>1<br>0.034<br>-
Ruskebukta (Norway)<br>n of pools<br>n of single samples<br>Sample conc.<br>n < LOD	69.20794;29.22895	 -<br>1<br>0.015<br>-	 -<br>1<br>0.39<br>-	 -<br>1<br>0.010<br>-	 -<br>1<br>0.035<br>-	 -<br>1<br>0.00033<br>-	 -<br>1<br>0.019<br>-	 -<br>1<br>0.040<br>-

Table S1.*Cont.*

	**Coordinates (Lat;Long)**	**Ni**	**Cu**	**Co**	**As**	**Cd**	**Pb**	**Hg**
Kuetsjarvi (Russia)<br>n of pools<br>n of fish in a pool<br>Sample conc.<br>n < LOD	69.46907;30.19638	1<br>5<br>0.16<br>-	1<br>5<br>0.12<br>-	1<br>5<br>0.039<br>-	1<br>5<br>0.012<br>-	1<br>5<br>0.0020<br>-	1<br>5<br>< 0.005<br>1	1<br>5<br>0.012<br>-
Rajakoski (Russia)<br>n of pools<br>n of fish in a pool<br>Sample conc.<br>n < LOD	69.02141;29.00452	1<br>5<br>< 0.005<br>1	1<br>5<br>0.091<br>-	1<br>5<br>0.0030<br>-	1<br>5<br>0.013<br>-	1<br>5<br>< 0.0005<br>1	1<br>5<br>0.0070<br>-	1<br>5<br>0.042<br>-
Virtuovoshjarvi (Russia)<br>n of pools<br>n of fish in a pool<br>Sample conc.<br>n < LOD	68.77037;28.82168	1<br>5<br>0.028<br>-	1<br>5<br>0.19<br>-	1<br>5<br>0.011<br>-	1<br>5<br>0.065<br>-	1<br>5<br>< 0.0005<br>1	1<br>5<br>0.012<br>-	1<br>5<br>0.11<br>-
Kochejaur (Russia)<br>n of pools<br>n of fish in a pool<br>Sample conc.<br>n < LOD	68.60266;28.6882	1<br>5<br>0.028<br>-	1<br>5<br>0.45<br>-	1<br>5<br>0.0050<br>-	1<br>5<br>0.025<br>-	1<br>5<br>< 0.0005<br>1	1<br>5<br>< 0.0005<br>1	1<br>5<br>0.14<br>-
Inari (Finland)<br>n of pools<br>n of fish in a pool<br>mean conc.<br>median conc.<br>min;max conc.<br>n < LOD	68.88994;27.62992	3<br>5<br>0.0093<br>0.007<br>0.005;0.016<br>-	-	-	-	-	-	-

Table S1.*Cont.*

	Coordinates (Lat;Long)	Ni	Cu	Co	As	Cd	Pb	Hg
**Perch**								
Tjærebukta (Norway)<br>n of pools<br>n of single samples<br>mean conc.<br>median conc.<br>min;max conc.<br>n < LOD	69.22725;29.18445	 -<br>2<br>0.0057<br>0.0057<br>0.0051;0.0063<br>-	 -<br>2<br>0.37<br>0.37<br>0.27;0.46<br>-	 -<br>2<br>0.0041<br>0.0041<br>0.0038;0.0044<br>-	 -<br>2<br>0.024<br>0.024<br>0.016;0.032<br>-	 -<br>2<br>0.00041<br>0.00041<br>0.00040;0.00042<br>-	 -<br>2<br>0.010<br>0.010<br>0.0077;0.013<br>-	 -<br>2<br>0.52<br>0.52<br>0.49;0.56<br>-
Rundvannet (Norway)<br>n of pools<br><br>n of fish in a pool<br>mean conc.<br>median conc.<br>min;max conc.<br>n < LOD	69.68162;30.07831	 4 + 1 single sample*<br>5<br>0.033<br>0.025<br><0.011;0.088<br>2	 4 + 1 single sample*<br>5<br>0.18<br>0.17<br>0.15;0.24<br>-	 4 + 1 single sample*<br>5<br>0.0086<br>0.0078<br>0.0045;0.015<br>-	 4 + 1 single sample*<br>5<br>0.019<br>0.019<br>0.016;0.021<br>-	 4 + 1 single sample*<br>5<br>0.0023<br>0.0027<br>0.0012;0.0030<br>-	 4 + 1 single sample*<br>5<br>0.0055<br>0.0053<br><0.0018;0.0097<br>1	 4 + 1 single sample*<br>5<br>0.29<br>0.23<br>0.044;0.86<br>-
Ruskebukta (Norway)<br>n of pools<br>n of single samples<br>Sample conc.<br>n < LOD	69.20794;29.22895	 -<br>1<br>0.016<br>-	 -<br>1<br>0.43<br>-	 -<br>1<br>0.0084<br>-	 -<br>1<br>0.013<br>-	 -<br>1<br>0.00082<br>-	 -<br>1<br>0.0060<br>-	 -<br>1<br>0.22<br>-
Kuetsjarvi (Russia)<br>n of pools<br>n of fish in a pool<br>Sample conc.<br>n < LOD	69.46907;30.19638	 1<br>5<br>0.035<br>-	 1<br>5<br>0.22<br>-	 1<br>5<br>0.011<br>-	 1<br>5<br>0.012<br>-	 1<br>5<br>0.0020<br>-	 1<br>5<br>0.0050<br>-	 1<br>5<br>0.080<br>-

Table S1. *Cont.*

	Coordinates (Lat;Long)	Ni	Cu	Co	As	Cd	Pb	Hg
Shuonojarvi (Russia)<br>n of pools<br>n of fish in a pool<br>Sample conc.<br>n < LOD	69.24621;30.01409	 1<br>5<br>0.020<br>-	 1<br>5<br>0.21<br>-	 1<br>5<br>0.0050<br>-	 1<br>5<br>0.013<br>-	 1<br>5<br>0.0020<br>-	 1<br>5<br>0.026<br>-	 1<br>5<br>0.23<br>-
Virtuovoshjarvi (Russia)<br>n of pools<br>n of fish in a pool<br>Sample conc.<br>n < LOD	68.77037;28.82168	 1<br>5<br>0.016<br>-	 1<br>5<br>0.23<br>-	 1<br>5<br>0.0060<br>-	 1<br>5<br>0.017<br>-	 1<br>5<br>0.0020<br>-	 1<br>5<br>0.024<br>-	 1<br>5<br>0.17<br>-
Kochejaur (Russia)<br>n of pools<br>n of fish in a pool<br>Sample conc.<br>n < LOD	68.60266;28.6882	 1<br>5<br>0.015<br>-	 1<br>5<br>0.26<br>-	 1<br>5<br>0.0030<br>-	 1<br>5<br>0.0040<br>-	 1<br>5<br>0.0010<br>-	 1<br>5<br>0.027<br>-	 1<br>5<br>0.37<br>-
Inari (Finland)<br>n of pools<br>n of fish in a pool<br>mean conc.<br>median conc.<br>min;max conc.<br>n < LOD	68.88994;27.62992	 2<br>5<br>0.045<br>0.045<br>0.0090;0.081<br>-	 3<br>5<br>0.27<br>0.22<br>0.21;0.37<br>-	 -	 3<br>5<br>0.014<br>0.0081<br>0.0069;0.0257<br>-	 3<br>5<br>0.00095<br>0.0010<br>0.00075;0.0011<br>-	 3<br>5<br>0.0056<br>0.006<br>< 0.005;0.0074<br>1	 3<br>5<br>0.065<br>0.056<br>0.039;0.10<br>-
Vastusjärvi (Finland)<br>n of pools<br>n of fish in a pool<br>Sample conc.<br>n < LOD	69.07503;27.13353	 -	 1<br>5<br>0.33<br>-	 -	 1<br>5<br>0.0043<br>-	 1<br>5<br>0.0027<br>-	 1<br>5<br>0.011<br>-	 1<br>5<br>0.057<br>-

Table S1. *Cont.*

	**Coordinates (Lat;Long)**	**Ni**	**Cu**	**Co**	**As**	**Cd**	**Pb**	**Hg**
**Pike**								
Tjærebukta (Norway)<br>n of pools<br>n of single samples<br>Sample conc.<br>n < LOD	69.22725;29.18445	 -<br>1<br>0.0042<br>-	 -<br>1<br>0.28<br>-	 -<br>1<br>0.0019<br>-	 -<br>1<br>0.019<br>-	 -<br>1<br>0.00026<br>-	 -<br>1<br>0.0027<br>-	 1<br>0.18<br>-
Ruskebukta (Norway)<br>n of pools<br>n of single samples<br>Sample conc.<br>n < LOD	69.20794;29.22895	 -<br>1<br>0.0042<br>-	 -<br>1<br>0.19<br>-	 -<br>1<br>0.0012<br>-	 -<br>1<br>0.079<br>-	 -<br>1<br>0.00024<br>-	 -<br>1<br>0.0084<br>-	 -<br>1<br>0.23<br>-
Kuetsjarvi (Russia)<br>n of pools<br>n of fish in a pool<br>Sample conc.<br>n < LOD	69.46907;30.19638	 1<br>5<br>0.053<br>-	 1<br>5<br>0.098<br>-	 1<br>5<br>0.0080<br>-	 1<br>5<br>0.027<br>-	 1<br>5<br>0.0010<br>-	 1<br>5<br>0.0060<br>-	 1<br>5<br>0.062<br>-
Rajakoski (Russia)<br>n of pools<br>n of fish in a pool<br>Sample conc.<br>n < LOD	69.02141;29.00452	 1<br>5<br>0.032<br>-	 1<br>5<br>0.20<br>-	 1<br>5<br>0.0010<br>-	 1<br>5<br>0.012<br>-	 1<br>5<br>< 0.0005<br>1	 1<br>5<br>0.026<br>-	 1<br>5<br>0.51<br>-
Virtuovoshjarvi (Russia)<br>n of pools<br>n of fish in a pool<br>Sample conc.<br>n < LOD	68.77037;28.82168	 1<br>5<br>0.061<br>-	 1<br>5<br>0.21<br>-	 1<br>5<br>0.0030<br>-	 1<br>5<br>0.0090<br>-	 1<br>5<br>< 0.0005<br>1	 1<br>5<br>0.0060<br>-	 1<br>5<br>0.099<br>-

Table S1. *Cont.*

	Coordinates (Lat;Long)	Ni	Cu	Co	As	Cd	Pb	Hg
Kochejaur (Russia)<br>n of pools<br>n of fish in a pool<br>Sample conc.<br>n < LOD	68.60266;28.6882	 1<br>5<br>0.016<br>-	 1<br>5<br>0.17<br>-	 1<br>5<br>0.0010<br>-	 1<br>5<br>0.0020<br>-	 1<br>5<br>0.0030<br>-	 1<br>5<br>< 0.005<br>1	 1<br>5<br>0.18<br>-
Inari (Finland)<br>n of pools<br>n of fish in a pool<br>Sample conc.<br>n < LOD	68.88994;27.62992	 1<br>4<br>0.0040<br>-	 1<br>4<br>0.20<br>-	 -	 1<br>4<br>0.14<br>-	 1<br>4<br>< 0.00025<br>1	 1<br>4<br>< 0.005<br>1	 1<br>4<br>0.14<br>-
**Arctic char**								
Durvatn (Norway)<br>n of pools<br>n of fish in a pool<br>Sample conc.<br>n < LOD	69.61076;30.68831	 1<br>10<br>0.059<br>-	 1<br>10<br>0.44<br>-	 1<br>10<br>0.038<br>-	 1<br>10<br>0.031<br>-	 1<br>10<br>0.0035<br>-	 1<br>10<br>0.0073<br>-	 1<br>10<br>0.16<br>-
Rabvatn (Norway)<br>n of pools<br>n of fish in a pool<br>Sample conc.<br>n < LOD	69.64263;30.46097	 1<br>8<br>0.088<br>-	 1<br>8<br>0.35<br>-	 1<br>8<br>0.020<br>-	 1<br>8<br>0.044<br>-	 1<br>8<br>0.0062<br>-	 1<br>8<br>0.18<br>-	 1<br>8<br>0.17<br>-
Holmvatn (Norway)<br>n of pools<br>n of fish in a pool<br>Sample conc.<br>n < LOD	69.7147;30.76745	 1<br>8<br>0.046<br>-	 1<br>8<br>0.40<br>-	 1<br>8<br>0.028<br>-	 1<br>8<br>0.0096<br>-	 1<br>8<br>0.0076<br>-	 1<br>8<br>0.037<br>-	 1<br>8<br>0.043<br>-

Table S1.*Cont.*

	**Coordinates (Lat;Long)**	**Ni**	**Cu**	**Co**	**As**	**Cd**	**Pb**	**Hg**
Shuonojarvi (Russia)<br>n of pools<br>n of fish in a pool<br>Sample conc.<br>n < LOD	69.24621;30.01409	 1<br>5<br>0.046<br>-	 1<br>5<br>0.51<br>-	 1<br>5<br>0.018<br>-	 1<br>5<br>0.025<br>-	 1<br>5<br>0.0030<br>-	 1<br>5<br>0.090<br>-	 1<br>5<br>0.049<br>-
**Brown trout**								
Durvatn (Norway)<br>n of pools<br>n of fish in a pool<br>Sample conc.<br>n < LOD	69.61076;30.68831	 1<br>10<br>0.097<br>-	 1<br>10<br>0.55<br>-	 1<br>10<br>0.057<br>-	 1<br>10<br>0.031<br>-	 1<br>10<br>0.0024<br>-	 1<br>10<br>0.0063<br>-	 1<br>10<br>0.074<br>-
Gardsjøen (Norway)<br>n of pools<br>n of fish in a pool<br>Sample conc.<br>n < LOD	69.70603;30.84589	 1<br>7<br>0.072<br>-	 1<br>7<br>0.32<br>-	 1<br>7<br>0.045<br>-	 1<br>7<br>0.026<br>-	 1<br>7<br>0.0033<br>-	 1<br>7<br>0.022<br>-	 1<br>7<br>0.097<br>-
Rabvatn (Norway)<br>n of pools<br>n of fish in a pool<br>Sample conc.<br>n < LOD	69.64263;30.46097	 1<br>10<br>0.11<br>-	 1<br>10<br>0.38<br>-	 1<br>10<br>0.027<br>-	 1<br>10<br>0.042<br>-	 1<br>10<br>0.0025<br>-	 1<br>10<br>0.037<br>-	 1<br>10<br>0.063<br>-
Holmvatn (Norway)<br>n of pools<br>n of fish in a pool<br>Sample conc.<br>n < LOD	69.7147;30.76745	 1<br>7<br>0.066<br>-	 1<br>7<br>0.32<br>-	 1<br>7<br>0.061<br>-	 1<br>7<br>0.025<br>-	 1<br>7<br>0.0027<br>-	 1<br>7<br>0.021<br>-	 1<br>7<br>0.14<br>-

Table S1. *Cont.*

	**Coordinates (Lat;Long)**	**Ni**	**Cu**	**Co**	**As**	**Cd**	**Pb**	**Hg**
Kuetsjarvi (Russia)	69.46907;30.19638							
n of pools		-	-	-	-	-	-	-
n of single samples		1	1	1	1	1	1	1
Sample conc.		0.012	0.672	0.004	0.012	< 0.0005	< 0.005	0.145
n < LOD		-	-	-	-	1	1	-
**Game**								
**Reindeer**								
Pasvik (Norway)	69.57331;29.83184							
n of pools		-	-	-	-	-	-	-
n of single samples		10	10	10	10	10	10	10
mean conc.		0.0095	2.1	0.0079	0.033	0.0013	0.0028	< 0.005
median conc.		0.0071	2.1	0.0076	0.032	0.0011	0.0025	
min;max conc.		0.0033;0.027	1.6;2.5	0.0052;0.011	0.025;0.048	0.00072;0.0034	0.0016;0.0046	
n < LOD		-	-	-	-	-	-	10
Paistunturi (Finland)	69.6641;26.28446							
n of pools		-	-	-	-	-	-	-
n of single samples		3	3		3	3	3	3
mean conc.		0.018	2.2		0.014	0.0047	0.0077	0.0042
median conc.		0.022	2.3		0.014	0.0046	0.0087	0.0039
min;max conc.		0.010;0.023	2.1;2.34		0.013;0.015	0.0042;0.0053	0.0050;0.0093	0.0039;0.0047
n < LOD		-	-		-	-	-	-
**Moose**								
Pasvik (Norway)	69.65713;30.28188							
n of pools		-	-	-	-	-	-	-
n of single samples		3	3	3	3	3	3	3
mean conc.		0.034	1.6	0.011	0.0076	0.0015	0.12	< 0.005
median conc.		0.035	1.4	0.011	0.0070	0.0015	0.027	
min;max conc.		< 0.011;0.059	1.2;2.0	0.0074;0.015	0.0039;0.012	0.0013;0.0018	0.0066;0.34	
n < LOD		1	-	-	-	-	-	3

Table S1.*Cont.*

	**Coordinates (Lat;Long)**	**Ni**	**Cu**	**Co**	**As**	**Cd**	**Pb**	**Hg**
Russia<br>n of pools<br>n of single samples<br>Sample conc.<br>n < LOD	Unknown	 -<br>1<br>0.0080<br>-	 -<br>1<br>1.3<br>-	 -<br>1<br>0.0090<br>-	 -<br>1<br>< 0.001<br>1	 -<br>1<br>0.0020<br>-	 -<br>1<br>0.019<br>-	 -<br>1<br>0.0030<br>-
Kyläjoki (Finland)<br>n of pools<br>n of single samples<br>Sample conc.<br>n < LOD	68.42504;26.64045	 -<br>1<br>0.014<br>-	 -<br>1<br>1.8<br>-	 -	 -<br>1<br>0.00060<br>-	 -<br>1<br>0.00070<br>-	 -<br>1<br>0.034<br>-	 -<br>1<br>0.0014<br>-
Angeli, Kaitavaara (Finland)<br>n of pools<br>n of single samples<br>Sample conc.<br>n < LOD	68.91099;25.65843	 <br>-<br>1<br>0.0030<br>-	 <br>-<br>1<br>1.4<br>-	 <br>-	 <br>-<br>1<br>0.00060<br>-	 <br>-<br>1<br>0.0019<br>-	 <br>-<br>1<br>0.010<br>-	 <br>-<br>1<br>0.0019<br>-
Kuttura, Köysivaara (Finland)<br>n of pools<br>n of single samples<br>Sample conc.<br>n < LOD	68.3717;26.58609	 <br>-<br>1<br>0.0050<br>-	 <br>-<br>1<br>1.6<br>-	 <br>-	 <br>-<br>1<br>0.00070<br>-	 <br>-<br>1<br>0.0011<br>-	 <br>-<br>1<br>0.0078<br>-	 <br>-<br>1<br>0.0022<br>-
**Ptarmigan**								
Sevettijärvi (Finland)<br>n of pools<br>n of birds in a pool<br>Sample conc.<br>n < LOD	69.50567;28.58221	 1<br>4<br>0.0070<br>-	 1<br>4<br>4.7<br>-	 -	 1<br>4<br>0.0030<br>-	 1<br>4<br>0.019<br>-	 1<br>4<br>< 0.005<br>1	 1<br>4<br>0.00066<br>-

Table S1.*Cont*.

	Coordinates (Lat;Long)	Ni	Cu	Co	As	Cd	Pb	Hg
Utsjoki (Finland)<br>n of pools<br>n of birds in a pool<br>mean conc.<br>median conc.<br>min;max conc.<br>n < LOD	69.78764;27.80848	2<br>2 and 3<br>0.0040<br>0.0040<br>0.0030;0.0050<br>-	2<br>2 and 3<br>5.2<br>5.2<br>5.2;5.2<br>-	-	2<br>2 and 3<br>0.0030<br>0.0030<br>0.0028;0.0031<br>-	2<br>2 and 3<br>0.0084<br>0.0084<br>0.0069;.0099<br>-	2<br>2 and 3<br>< 0.005<br><br><br>2	2<br>2 and 3<br>0.00059<br>0.00059<br>0.00047;0.0007<br>-

(Lat = latitude, Long = longitude, Ni = nickel, Cu = copper, Co = cobalt, As = arsenic, Cd = cadmium, Pb = lead, Hg = total mercury, n = number of pools or samples in a pool, min = minimum, max = maximum, < LOD = concentrations below level of detection, ref. = reference site, conc. = concentration). Coordinates are approximate since some are for lakes and grazing regions. Concentrations below LOD were substituted with a numeric value (LOD/√2). These values are included in the calculation of the mean and median.
* 4 pools with 5 individual fish in each pool and one single sample

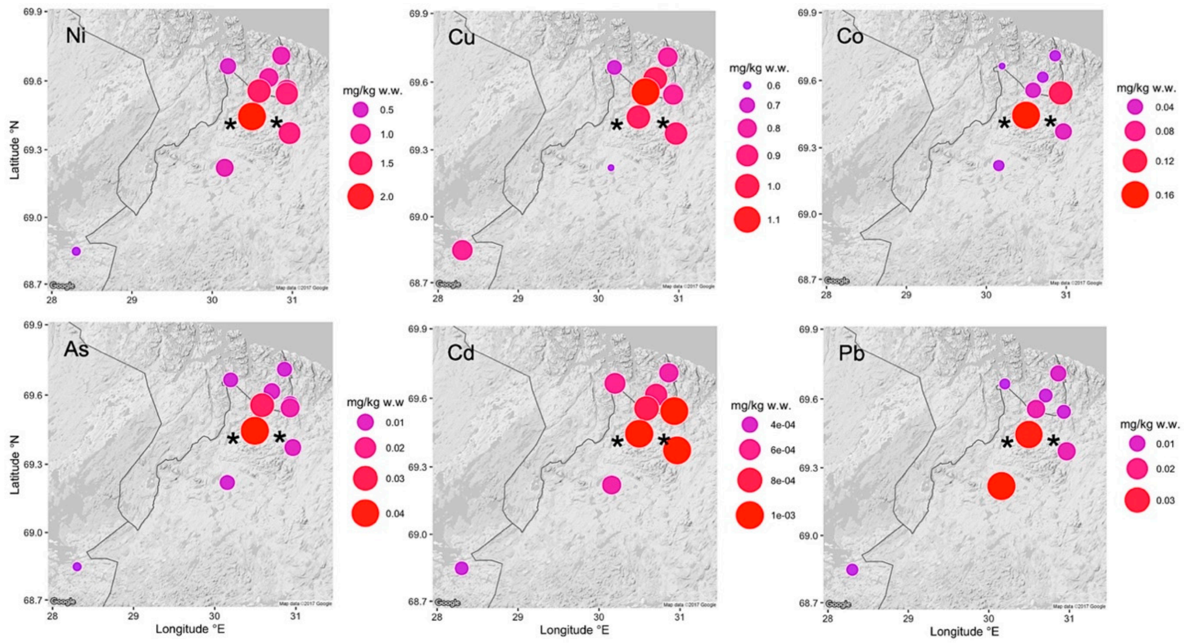

Figure S1. Concentration (mg/kg w.w.) of toxic elements in lingonberries in the border region of Norway, Finland and Russia. The cities Nikel and Zapolyarny are indicated by black stars. Note that concentration scales are different for the different elements.

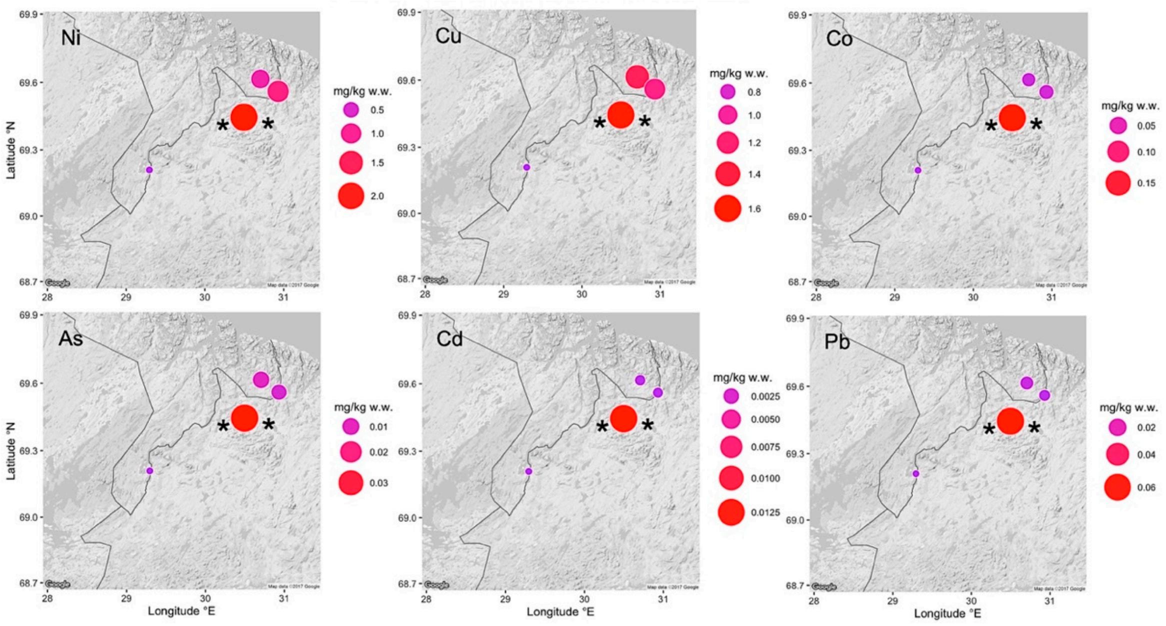

Figure S2. Concentration (mg/kg w.w.) of toxic elements in crowberries in the border region of Norway, Finland and Russia. The cities Nikel and Zapolyarny are indicated by black stars. Note that concentration scales are different for the different elements.

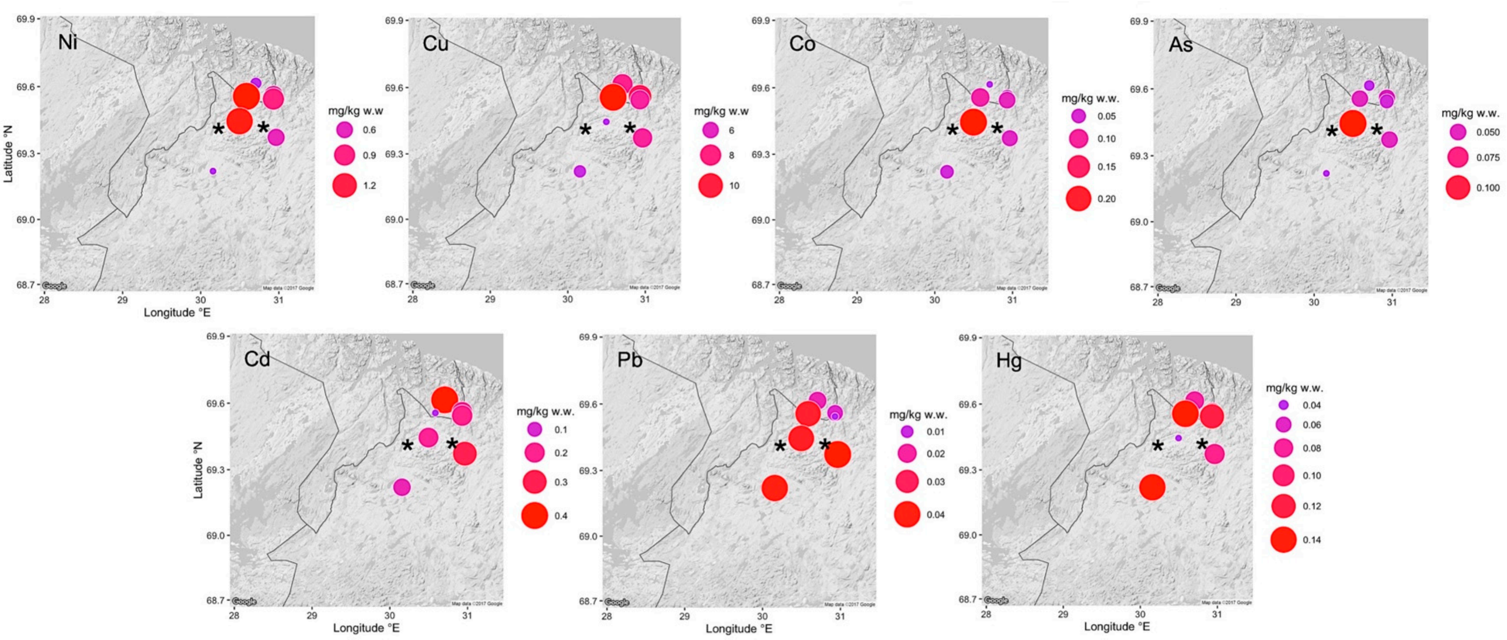

Figure S3. Concentration (mg/kg w.w.) of toxic elements in mushrooms (*Leccinum* genus) in the border region of Norway, Finland and Russia. The cities Nikel and Zapolyarny are indicated by black stars. Note that concentration scales are different for the different elements.

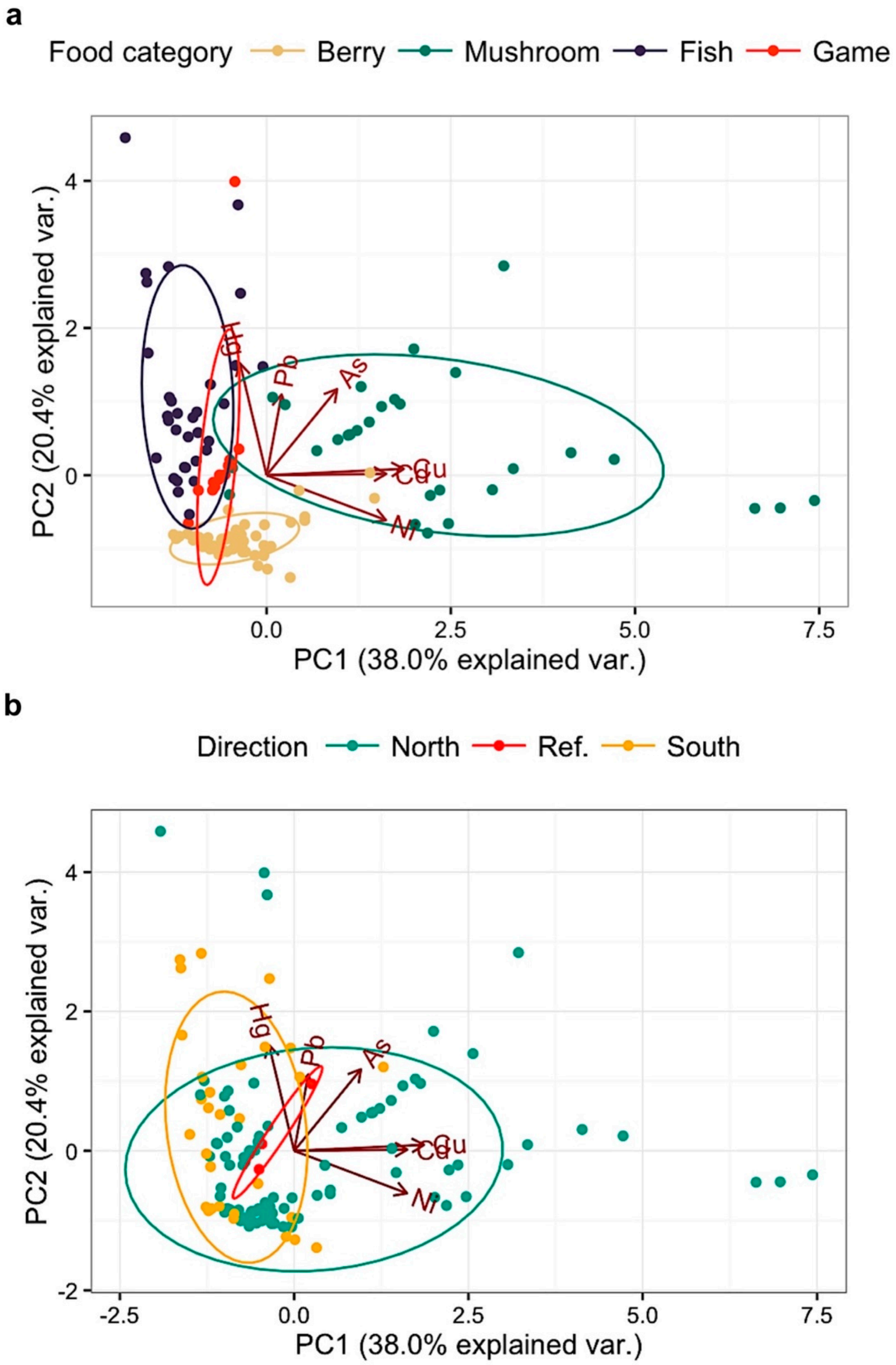

Figure S4. Principal component biplots of **(a)** food groups (berries: yellow, mushrooms: green, fish: purple, and game: red) and **(b)** sample location north of the Ni-Cu smelter (north: green), south of the smelter (south: yellow) or at reference sites (ref.: red) in the projection of principal component 1 (38.0 % explained variance) and principal component 2 (20.4 % explained variance).

Table S2. European Union maximum levels for toxic elements in food items [36-39] compared with results from the present study. w.w. = wet weight.

Maximum levels set by the EU		Observed data			
		**Species**	**Location**	**n > ML / n_s ***	**Range of samples > ML**
**Lead (mg/kg w.w.):**					
Fish muscle	0.30	None exceeded			
Meat †	0.10	Moose	Pasvik (Norway)	1 / 11	0.34
		Ptarmigan	Russia	1 / 4	0.17
Berries and small fruit	0.20	None exceeded			
**Cadmium (mg/kg w.w.)**					
Meat †	0.050	None exceeded			
Fish muscle	0.050	None exceeded			
Fungi	1.0	Gypsy mushroom	Gardsjøen (Norway)	3 / 12	1.1 - 1.5
			Durvatn (Norway)	3 / 12	1.3 - 1.9
**Mercury (mg/kg w.w.)**					
Fish muscle ‡	0.50	Perch	Tjærebukta (Norway)	1 / 16	0.56
			Rundvannet (Norway)	1 / 16	0.86
Pike muscle	1.0	None exceeded			

* n > ML / n_s = number of samples exceeding maximum levels out of the total number of samples of the same species

† of bovine animals, sheep, pig and poultry (excluding offal)

‡ and fishery products
